# Path Planning for Unmanned Delivery Robots Based on EWB-GWO Algorithm

**DOI:** 10.3390/s23041867

**Published:** 2023-02-07

**Authors:** Yuan Luo, Qiong Qin, Zhangfang Hu, Yi Zhang

**Affiliations:** 1 Key Laboratory of Optoelectronic Information Sensing and Technology, Chongqing University of Posts and Telecommunications, Chongqing 400065, China; 2 School of Advanced Manufacturing Engineering, Chongqing University of Posts and Telecommunications, Chongqing 400065, China

**Keywords:** mobile robot, GWO algorithm, path planning, two-dimensional chaotic mapping, adaptive nonlinear inertia weights, BOA algorithm, opposition-based learning

## Abstract

With the rise of robotics within various fields, there has been a significant development in the use of mobile robots. For mobile robots performing unmanned delivery tasks, autonomous robot navigation based on complex environments is particularly important. In this paper, an improved Gray Wolf Optimization (GWO)-based algorithm is proposed to realize the autonomous path planning of mobile robots in complex scenarios. First, the strategy for generating the initial wolf pack of the GWO algorithm is modified by introducing a two-dimensional Tent–Sine coupled chaotic mapping in this paper. This guarantees that the GWO algorithm generates the initial population diversity while improving the randomness between the two-dimensional state variables of the path nodes. Second, by introducing the opposition-based learning method based on the elite strategy, the adaptive nonlinear inertia weight strategy and random wandering law of the Butterfly Optimization Algorithm (BOA), this paper improves the defects of slow convergence speed, low accuracy, and imbalance between global exploration and local mining functions of the GWO algorithm in dealing with high-dimensional complex problems. In this paper, the improved algorithm is named as an EWB-GWO algorithm, where EWB is the abbreviation of three strategies. Finally, this paper enhances the rationalization of the initial population generation of the EWB-GWO algorithm based on the visual-field line detection technique of Bresenham’s line algorithm, reduces the number of iterations of the EWB-GWO algorithm, and decreases the time complexity of the algorithm in dealing with the path planning problem. The simulation results show that the EWB-GWO algorithm is very competitive among metaheuristics of the same type. It also achieves optimal path length measures and smoothness metrics in the path planning experiments.

## 1. Introduction

**Figure 1:**
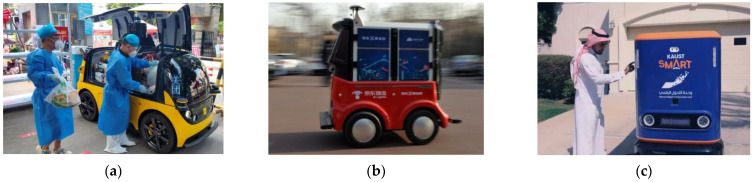
Unmanned delivery robots enable fast, efficient, and contactless delivery services. (**a**) Meituan delivery robots deliver living supplies to epidemic-stricken areas; (**b**) JD delivery robot cargo transportation test; (**c**) UISEE delivery robot enables unmanned delivery service at KAUST.

Initially, mobile robots were widely used in high-risk areas in industrial and military industries. Unmanned Aerial Vehicles (UAVs) [1] and Autonomous Underwater Vehicles (AUVs) [2] have gradually become the essential backbone of national armies due to their lower cost of use, easy logistical support, and lower safety risk factor. Rail-Guided Vehicles (RGVs) [3] and Automated Guided Vehicles (AGV) [4] are becoming an indispensable part of the automation industry chain in the industrial field due to their advantages of simple and convenient operation, strong autonomy, and high work efficiency. With the rise of Industry 4.0 and emerging technologies, mobile robots are playing an important role in search and rescue [5], cargo transportation [6], unmanned services [7], geological exploration, and cultural entertainment [8]. Advances in autonomous navigation technology for mobile robots have made it possible to build unmanned delivery networks on the ground. The unmanned delivery robot developed by JD entered the road test phase in 2016 and will be put into operation soon. The technology makes it possible to achieve a large-scale and contactless delivery service of goods during an epidemic to meet the livelihood needs of a large number of consumers who are unable to travel, as shown in Figure 1. Currently, unmanned delivery robots are still severely limited in urban areas, especially in heavy and congested traffic environments. A significant portion of delivery robots are limited to areas with more fixed environments, such as campuses and communities. This still does not take advantage of unmanned delivery robots to replace people in the current epidemic environment for efficient, fast, and contactless delivery services. Therefore, the reasonable construction of unmanned distribution networks, the large-scale application of mobile robots for efficient cargo transportation, and the solution of the problem of “the last mile of cargo delivery” will bring great convenience to consumers.

Unmanned delivery robots are not the same as traditional AGVs in terms of navigation methods, movement modes, and usage functions. Traditional AGVs are based on a differential drive model with radar and laser SLAM navigation [9]. AGVs move slowly and have a lifting capacity of tons, enabling factories to move heavyweight goods while meeting the needs of distribution safety. Although unmanned delivery robots have fast braking, GPS, and a vehicle motion model design structure to enhance the adaptability of road transportation to some extent, they still face serious challenges in the delivery tasks of large and complex maps.

Path planning, as one of the key technologies for the autonomous navigation of unmanned delivery robots, aims to achieve a short, efficient, and safe walking route for mobile robots to quickly plan a path from the initial position to the target area in complex work scenarios. In the new industrial era, with the introduction of intelligent industries, the operational efficiency of the current robot path planning algorithm and the quality of the generated path no longer meet the requirements of the times. For mobile robots performing unmanned delivery work in large-scale complex environments, the path planning of mobile robots is a key factor affecting work efficiency. Therefore, to improve the efficiency of delivery robot operations, it is necessary to shorten the path length and reduce the running time of the path planning algorithm while satisfying the path feasibility and autonomous obstacle avoidance of the mobile robot. The authors in [10] state that traditional path planning algorithms for mobile robots lead to an exponential increase in planning difficulty when dealing with complex environments with a large number of uncertainties. Finding a path between two nodes that satisfies the constraints of the mobile robot is understood as an NP problem, and there is no universal solution. Therefore, in this paper, in order to solve the above problem, an improved metaheuristic intelligent search algorithm is introduced to abstract the path planning problem of the delivery robot into an optimization problem with movement space constraints. Efficient autonomous path planning for unmanned delivery robots in complex environments with large maps is achieved through the ability of the metaheuristic algorithm to handle highly nonlinear problems.

## 2. Related Work

In recent years, many scholars have researched and developed path planning techniques for mobile robots under complex maps in order to guarantee the safety and efficiency of robots in the process of movement.

The graph search technique of grid discretization [11] is popular as one of the most intuitive computational processes among path planning algorithms for its simplicity of operation, ease of map construction, and flexibility of map discretization intervals. In the article [12], weighting methods for three different strategies, weight-based recurrence (WBR), weight-based coordinate distance (WBCD), and weight-based travel cost (WBTC), are proposed and fused with heuristic functions of A*, Bi-A*, and Jump Point Search (JPS) algorithms to further improve the computational efficiency of path planning algorithms based on discrete grid map search techniques. It is a simple and effective way to reduce the computational complexity of the algorithm by adding weights to strengthen the guidance of the path planning algorithm in the search process, reducing the detection of redundant nodes by the algorithm. However, the efficiency of the algorithm will decrease significantly as the density of obstacles and the complexity of the map increase, and the limitation of the search direction of the algorithm due to the discretized grid will make the generated path of the algorithm differ from the actual optimal path [13]. In the article [14], a Theta* algorithm based on selective parameter optimization is proposed, and an operational trajectory conforming to the kinematic constraints of the vehicle is accomplished by eliminating the path-redundant nodes. The Theta* algorithm [15] adds an any-angle pathfinding strategy to the traditional A* algorithm and prunes the redundant nodes in the path through line-of-sight (LOS) reachability detection [16,17] to achieve the purpose of shortening the path. However, it also has the disadvantage that the time complexity of the algorithm is too large and not conducive to the efficient and fast execution of delivery tasks by mobile robots.

Path planning algorithms based on probabilistic sampling techniques usually require sampling the nodes of the mobile robot’s workspace in a random way and finding the optimal path by controlling the search direction and search step of the algorithm. The authors in [18] proposed a Rapidly exploring Random Trees (RRT) path planning algorithm based on low-cost valley and cost-space saddle-point strategies, which biases the random selection of path nodes to low-cost regions in space to achieve the low-cost motion of the mobile robot. The path planning algorithm based on the probabilistic sampling technique reduces to some extent the limitation of the search direction caused by the discretized grid map, but it is worth noting that the path planning algorithm based on sampling technique provides only weak probabilistic completeness and the generated paths are not optimal in general. Based on the above problems, [19] proposed a pseudo-random sampling strategy based on spatial principal axis as reference to further optimize the path planning method based on the sampling technique. This article improves the computational efficiency of the algorithm and the quality of the generated paths by setting the distance threshold between nodes and using a two-way incremental approach for path collision detection. In [20], a neural RRT* algorithm is proposed to guide the sampling location of RRT* and speed up the convergence of the algorithm by training the convolutional neural network (CNN) model for a large number of successful path planning cases for a more accurate prediction of the probability distribution of the optimal path.

The ability of intelligent search techniques based on metaheuristic algorithms to solve highly nonlinear and complex problems has led to widespread interest in their use in the field of path planning. The authors in [21] presented a path planning method based on an improved particle swarm optimization (PSO) algorithm. This paper solves the shortcomings of the PSO algorithm by adding adaptive fractional-order velocity to the iterative process of the PSO algorithm and applying local perturbation to the particle swarm through the evolutionary state of the PSO algorithm to solve the shortcomings of the PSO algorithm, which is prone to fall into a local optimum and premature maturity of the algorithm, and thus realizes the path planning design of mobile robots. The authors in [22] proposed a Mayfly (MF) algorithm based on an adaptive Cauchy variational operator and exponentially decreasing inertia weighting strategy to achieve two-dimensional autonomous pathfinding for UAVs. In addition to perfect algorithmic scalability in finding high-quality solutions to many complex problems, metaheuristics are favored by many researchers for their ability to excel when traditional exact optimization algorithms fail to provide satisfactory results. In 2014, Seyedali Mirjalili proposed the Gray Wolf Optimization (GWO) algorithm in his article [23], which is based on the multilayer social structure of gray wolf packs and uses the social behavior of wolves to find and round up prey to determine the best location for rounding up prey. The results of the benchmark function tests in the article show that the GWO algorithm is more competitive with other metaheuristics of the same type. However, according to no-free-lunch theorem for optimization theory [24], it is known that no optimization algorithm can outperform any other algorithm under any metric for all possible problems, and the same is true for the GWO algorithm. The authors in [25] pointed out that the GWO algorithm suffers from the defects of the slow convergence of the algorithm and an easy to fall into local optimum, resulting in a premature algorithm and the poor progress of optimization results when dealing with high-dimensional complex functions.

In recent years, many scholars have proposed many optimization strategies for the GWO algorithm with the intention of further improving the search efficiency, convergence accuracy, and performance balance of the GWO algorithm. Article [26] proposed an improved GWO algorithm (I-GWO) based on the dimensional-learning hunting search (DLH) strategy, which inherits the hunting behavior of individual wolves in nature. The DLH strategy uses a different approach to construct a hunting domain for each wolf, in which neighboring information can be shared among individual wolves to enhance the performance balance between local and global search capabilities and further maintain the diversity of the population. The DLH strategy enables the I-GWO algorithm to have excellent global convergence and local optimal escape capability. Article [27] proposed a hybrid algorithm GWO-CS based on the Cuckoo Search (CS) algorithm and the GWO algorithm. The article further optimizes the global search process of the GWO algorithm by using the location update formula of the CS algorithm. It optimizes the strongly oriented global search process in the GWO algorithm by using the random wandering of the CS algorithm and the global search method of Lévy flight to update the nest location, and improves the local optimal stagnation and prematureness of the algorithm due to the weak global search performance of the GWO algorithm. Article [28] proposed an exponential convergence factor improvement strategy to better fit the actual search process of gray wolves, improving the control parameters to balance the global exploration and local exploitation capabilities of the algorithm while incorporating dynamic weighting factors to reduce the possibility of the GWO algorithm falling into a local optimum. However, from the experimental results, the GWO algorithm still has the problem of the low accuracy of optimization results when dealing with high-dimensional functions.

The GWO algorithm is also often used in the process of metaheuristic algorithm improvement and fusion to solve the parameter setting of other kinds of metaheuristic algorithms and selective optimization problems in the running process. Article [29] proposed a hybrid algorithm combining PSO and GWO. The algorithm is not designed with the same idea as GWO-CS. The article is developed without changing the general operation of the PSO algorithm and GWO algorithm. The authors use the excellent detection capability of the GWO algorithm to replace the randomness drift of particles in the original PSO algorithm by directing some particles to the partially improved positions obtained by the GWO algorithm, further preventing the PSO algorithm from falling into the risk of the local optimal inability to escape; however, it is undeniable that the binary heuristic algorithm will bring a surge in time complexity. The paper points out that the increase in the time complexity of the algorithm is tolerable from a practical engineering point of view, such as the leather nesting problem (LNP).

Based on the above problems and inspired by related works, this paper improves the path planning design of unmanned delivery robots by improving the GWO algorithm to ensure the safe and efficient pathfinding of the robots in large and complex map scenarios. The contributions made in this paper are shown below.

1. The strategy of generating the initial wolf pack based on the GWO algorithm is modified by introducing a two-dimensional Tent–Sine coupled chaotic mapping based on the two-dimensionality of the mobile robot path solution. Improving the randomness between the two-dimensional state variables (*x*-axis coordinate positions and *y*-axis coordinate positions) of the path nodes while guaranteeing the population diversity of the generated GWO algorithm;
2. An improved GWO algorithm (EWB-GWO) is proposed, which combines an adaptive nonlinear inertia weighting strategy, opposition-based learning method based on an elite strategy, and a random wandering law of the BOA algorithm to balance the search performance of the GWO algorithm and improve the convergence accuracy of the algorithm;
3. The EWB-GWO algorithm is combined with mobile robot path planning to enhance the rationalization of the initial population generation through the line-of-sight detection technique based on Bresenham’s line algorithm, reduce the number of iterations of the EWB-GWO algorithm, and decrease the time complexity of the algorithm;
4. The EWB-GWO algorithm and its three sub-algorithms are tested against other metaheuristics (PSO, GA, BOA, ABC) based on the CEC benchmark function test set in a cross-sectional comparison, and the results are analyzed to further validate the rationality of the algorithm improvement strategy;
5. Experiments on mobile robot path planning based on the public dataset of Moving AI Lab and a cross-sectional comparison with other path planning algorithms to verify the superiority of EWB-GWO algorithm in handling large-scale complex environment maps.

## 3. Gray Wolf Optimization Algorithm and Its Improvement Strategy

The GWO algorithm is a swarm intelligence (SI) search algorithm among the natural metaheuristics, which is inspired by the roundup and hunting behavior of the gray wolf pack. Three high-ranking gray wolf individuals lead the pack in the optimal direction of the prey, which makes the method a successful algorithm for fast convergence and avoiding local minima [25]. However, the GWO algorithm suffers from the same lack of population diversity, an uneven exploration and exploitation strategy, and the premature convergence of the algorithm under high-dimensional complexity functions. Next, this paper will give the GWO algorithm and the detailed steps to change the method.

### 3.1. Standardized GWO Algorithm

**Figure 2:**
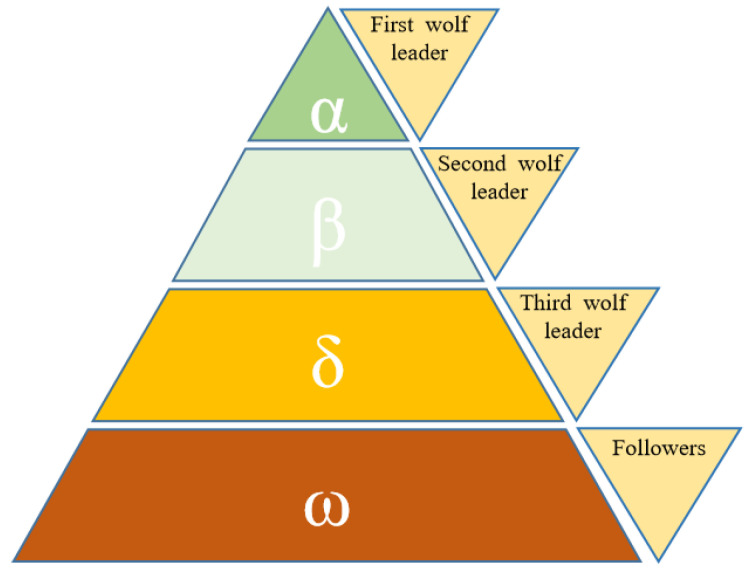
Hierarchy of the Gray Wolf Pack (dominance decreases from top down).

The GWO algorithm was proposed by Seyedali Mirjalili, an academic at Griffith University, Australia, and others, and was inspired by the hunting activity behavior of gray wolf packs. The GWO algorithm mathematically simulates how a gray wolf pack searches, surrounds and attacks its prey, and strictly follows the social hierarchy of the pack, with three leading wolves leading the overall action of the pack. The social hierarchy of the gray wolf pack divides the wolves into four different species, which play a role of mutual dominance in the hierarchy. Among them, Alpha (α) wolves, Beta (β) wolves, and Delta (δ) wolves are considered as pack leaders with excellent leadership ability, while Omega (ω) wolves are subordinate wolves following the leaders, as shown in Figure 2.

A mathematical modeling of the social hierarchy of wolves is required in the design of the GWO algorithm. Usually, during the iteration of the algorithm, the best solution is considered to be Wolf α, the second and third best solutions are Wolf β and Wolf δ, respectively, while all other alternatives are considered to be ω. During the search and exploration tasks performed by the GWO algorithm, the overall wolf pack’s search direction is dominated by α, β, and δ, while ω follows. The gray wolf is divided into three main stages in the hunting process: finding the target and approaching the prey, surrounding the prey and harassing it until the target stops moving, and finally attacking the prey.

#### 3.1.1. Approaching and Encircling Behavior of Wolves towards Their Prey

Gray wolves will surround the prey in the hunting process, and when the current position of the gray wolf pack is expressed as $\vec{X}(t)$ and the position information of the prey is expressed as $\vec{X_{p}}(t)$, the hunting behavior of the gray wolf pack is abstracted into a mathematical model, as shown in Equations (1) and (2)

(1) $$\vec{D}=|\vec{C}\cdot \vec{X_{p}}(t)-\vec{X}(t)|$$

(2) $$\vec{X}(t+1)=\vec{X_{p}}(t)-\vec{A}\cdot \vec{D},$$

where $\vec{D}$ denotes the distance between the prey and the wolf individuals and t is the number of current iterations. In Equation (2), $\vec{X}(t+1)$ represents the location update information of the gray wolf pack, and the movement of the pack will be affected by its distance from the current prey. $\vec{C}$ and $\vec{A}$ in the formula indicate the vector coefficients, whose expressions are shown specifically in Equations (3) and (4).

(3) $$\vec{A}=2\vec{a}\cdot \vec{r_{1}}-\vec{a}$$

(4) $$\vec{C}=2\cdot \vec{r_{2}}$$

Among them, the parameter $\vec{a}$ plays a role in the GWO algorithm to balance global search with local mining. The value of $\vec{a}$ decreases linearly from 2 to 0 during the overall iteration of the algorithm. A larger value of $\vec{a}$ in the initial stage of the algorithm iteration helps the global convergence of the GWO algorithm and guides the wolf pack quickly to the region where the optimal solution is located. The value of $\vec{a}$ decreases linearly in the later iterations of the algorithm, which helps the GWO algorithm to mine finely in the optimal region and improve the convergence accuracy of the algorithm. $\vec{r_{1}}$ and $\vec{r_{2}}$ are random vectors satisfying $\vec{r_{1}},\vec{r_{2}}\in [0,1]$. From Equations (1) and (2), it is known that the gray wolf can make a prediction of its moving direction based on the current position information and the possible orientation of the prey. By adjusting the values of variables $\vec{A}$ and $\vec{C}$ in Equations (1) and (2), the gray wolf can reach different locations around the prey to achieve encirclement.

#### 3.1.2. Hunting Behavior of Wolves

**Figure 3:**
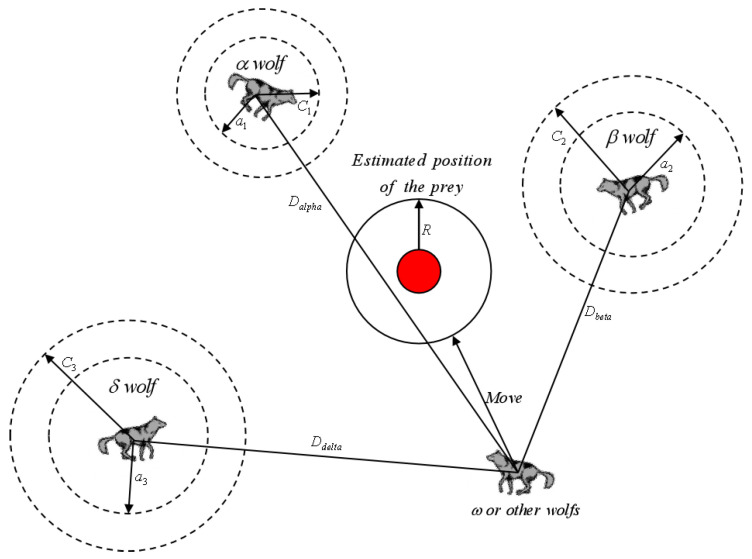
Position update of wolf groups in GWO algorithm.

The gray wolf pack has the ability to identify the location of potential prey and mainly relies on the guidance of wolf α, wolf β, and wolf δ in the process of searching for prey. In order to simulate the hunting behavior of gray wolves at the mathematical level, the GWO algorithm needs to keep the three wolves (α, β, and δ) with the best adaptation in the current pack during each iteration and update the positions of other wolves based on their position information. The mathematical model of wolf pack leaders tracking prey is represented as shown in Equation (5).

(5) $$\left\{\begin{array}{c} \vec{D_{\alpha }}=|\vec{C_{1}}\cdot \vec{X_{\alpha }}-\vec{X}|\\ \vec{D_{\beta }}=|\vec{C_{2}}\cdot \vec{X_{\beta }}-\vec{X}|\\ \vec{D_{\delta }}=|\vec{C_{3}}\cdot \vec{X_{\delta }}-\vec{X}| \end{array}\right.$$

where $\vec{D_{\alpha }}$, $\vec{D_{\beta }}$, and $\vec{D_{\delta }}$ denote the distance difference between wolf α, wolf β, and wolf δ and other individuals, respectively. $\vec{X_{\alpha }}$, $\vec{X_{\beta }}$, and $\vec{X_{\delta }}$ denote the current locations of wolf α, wolf β, and wolf δ, respectively, $\vec{X}$ denotes the current wolf pack location, and $\vec{C_{1}}$, $\vec{C_{2}}$, and $\vec{C_{3}}$ are random vectors and satisfy the constraints of Equation (4). Based on the distance difference between the wolf leader and the wolf pack, the traction direction of the overall wolf pack, i.e., the information on the moving orientation of the wolf pack, can be calculated as shown in Equations (6) and (7).

(6) $$\left\{\begin{array}{c} \vec{X_{1}}=\vec{X_{\alpha }}-\vec{A_{1}}\cdot (\vec{D_{\alpha }})\\ \vec{X_{2}}=\vec{X_{\beta }}-\vec{A_{2}}\cdot (\vec{D_{\beta }})\\ \vec{X_{3}}=\vec{X_{\delta }}-\vec{A_{3}}\cdot (\vec{D_{\delta }}) \end{array}\right.$$

(7) $$\vec{X}(t+1)=\frac{\vec{X_{1}}+\vec{X_{2}}+\vec{X_{3}}}{3}$$

where $\vec{A_{1}}$, $\vec{A_{2}}$, and $\vec{A_{3}}$ are random vectors satisfying the constraints of Equation (3); $\vec{X_{1}}$, $\vec{X_{2}}$, and $\vec{X_{3}}$ denote the traction directions of the three leading wolves; and $\vec{X}(t+1)$ denotes the overall moving position of the wolf pack. As shown in Figure 3, it can be seen that the final orientation of the wolves will be located at a random position within the circle defined by the positions of α, β, and δ in the search space.

Now, this paper shows the calculation steps of the basic Gray Wolf Optimization algorithm and the pseudo-code as follows (see Algorithm 1).

1. Initialize the number of wolf pack individuals (Pop), the number of iterations of the algorithm (MaxIter), the dimension of the solution space (Dim), and the boundary conditions (ub,lb) in the GWO algorithm;
2. Randomly generate the initial population of gray wolves in the solution space, then calculate the fitness value of each individual and initialize to Alpha, Beta, and Delta wolves according to the fitness function;
3. Calculate the distance between the wolf pack and the leading wolf according to Equation (5), and obtain the migration direction of the wolf pack according to Equation (6);
4. Re-update Alpha, Beta, and Delta wolves based on migrated wolf individuals;
5. If the number of iterations of the algorithm reaches the termination condition, output the order cycle and the optimal individual, otherwise update the parameter vector $\vec{a}$ and return to Step 3.

**Algorithm 1** Pseudocode for GWO algorithm 

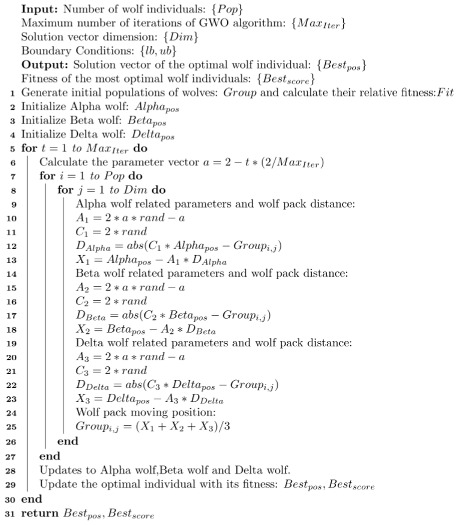



### 3.2. EWB-GWO Algorithm

The GWO algorithm achieves superiority-seeking by leading the wolf pack to the prey region during the iterative process, which makes the GWO algorithm have more efficient and fast convergence properties than other metaheuristic algorithms in dealing with high-dimensional complex optimization problems. However, as the complexity and dimensionality of the problem being solved increases, the convergence speed and accuracy of the GWO algorithm decreases significantly. At the same time, the GWO algorithm still has the disadvantage of low population diversity and an easy to fall into local optimum when dealing with complex multimodal functions. Therefore, in this paper, the GWO algorithm is modified by using Tent–Sine two-dimensional coupled chaotic mapping and combining three improvement strategies.

#### 3.2.1. Tent–Sine Two-Dimensional Coupled Chaotic Mapping

The generation of the initial population is usually an important factor to be considered by the GWO algorithm in solving optimization problems. In general, a random approach is used to generate the initial population; however, this approach can easily result in an uneven distribution of the initial solutions and distributed aggregation, making the search capability of the algorithm poor. Chaotic mappings are used to generate chaotic sequences, and their characteristics of nonlinearity, insensitivity to initial values, and high randomness can help the GWO algorithm to generate initial solutions and further maintain the diversity of populations.

**Figure 4:**
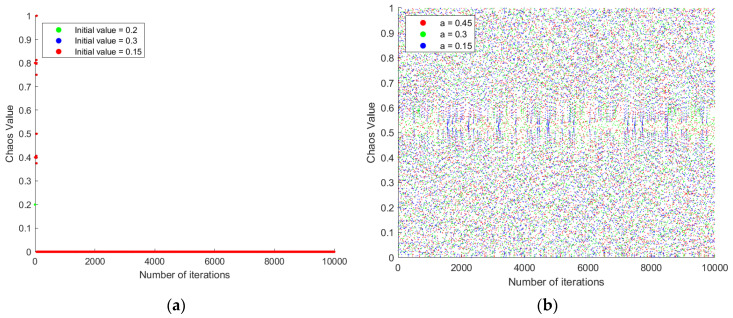
The small period nature of Tent chaotic mapping. (**a**) At *a* = 0.5 and initial values of 0.2, 0.4, and 0.8, the Tent chaotic mapping falls into a small loop, resulting in a sequence that is not chaotic in nature. (**b**) The random sequence generated by the Tent chaos mapping with *a* = 0.45 and initial values of 0.2, 0.3, and 0.15.

Tent mapping, also known as tent mapping, produces a random sequence with uniform probability distribution density and data correlation, and the mathematical expression is shown in Equation (8).

(8) $$x_{k+1}=\left\{\begin{array}{cc} \frac{x_{k}}{a} & x_{k}\in [0,a)\\ \frac{\left(1-x_{k}\right)}{1-a} & x_{k}\in [a,1] \end{array}\right.$$

when a=0.5, it is called the central Tent mapping, and the mapping sequence obtained at this time has a uniform distribution characteristic. However, according to Equation (8), when a=0.5, the Tent mapping will inevitably have a short-period characteristic in the iterative sequence, for example, when $x_{k}=0.2$, it will lead to $x_{k+1}=0.4$, $x_{k+2}=0.8$, and $x_{k+3}=0.2$, and thus fall into a cycle. This is shown in Figure 4. In order to jump out of the small cycle and form a two-dimensional initial population that satisfies the mathematical model of path planning, this paper uses a two-dimensional coupled Sine mapping approach to improve the Tent chaotic mapping.

**Figure 5:**
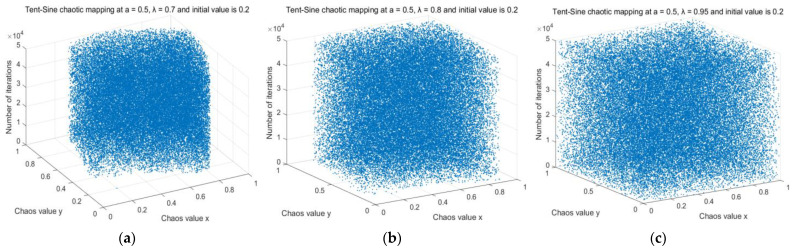
Distribution of generated sequences for Tent–Sine chaotic mapping with different λ parameters. (**a**) Tent–Sine chaotic mapping at *a* = 0.5, λ = 0.7, and initial value of 0.2. (**b**) Tent–Sine chaotic mapping at *a* = 0.5, λ = 0.8, and initial value of 0.2. (**c**) Tent–Sine chaotic mapping at *a* = 0.5, λ = 0.95, and initial value of 0.2.

The Sine mapping is a single-peaked chaotic mapping with a value range between −1 and 1. The mathematical expression of the Sine mapping is shown in Equation (9).

(9) $$\begin{array}{cc} x_{k+1}=b\sin(\pi x_{k}) & b\in \left(0,1\right] \end{array}$$

where b is the control parameter and the highest randomness of the resulting chaotic sequence as b approaches 1. However, the sequence generated by a single Sine mapping has sinusoidal properties, resulting in a low equilibrium of the generated random solutions. In this paper, the two mapping methods are coupled and the sequence generated by the Tent mapping is forced to achieve a cycle escape by the small perturbation provided by the Sine mapping, as shown in Equations (10) and (11).

(10) $$x_{k+1}=\left\{\begin{array}{ll} (\lambda \frac{x_{k}}{a}+(1-\lambda )\sin(\pi y_{k}))\; & x_{k}\in \left[0,a\right)\\ \left(\lambda \frac{1-x_{k}}{1-a}+(1-\lambda )\sin(\pi y_{k})\right) & x_{k}\in \left[a,1\right) \end{array}\right.$$

(11) $$y_{k+1}=\left\{\begin{array}{ll} \left(\lambda \frac{y_{k}}{a}+(1-\lambda )\sin(\pi x_{k+1})\right) & x_{k}\in \left[0,a\right)\\ \left(\lambda \frac{1-x_{k}}{1-a}+(1-\lambda )\sin(\pi x_{k+1})\right) & x_{k}\in \left[a,1\right) \end{array}\right.$$

where λ is the system parameter and satisfies λ∈(0,1). From Equations (10) and (11), it can be seen that the two-dimensional sequence $x_{k+1}$ and $y_{k+1}$ have a strong coupling property between them, and the two-dimensional sequence generated when a=0.5 and λ tends to 1 has a good uniformity distribution, as shown in Figure 5.

#### 3.2.2. Adaptive Nonlinear Inertia Weights

Among metaheuristic algorithms, inertia weights can be used to regulate the balance of the global search and local mining ability of the algorithm, which has a crucial impact on the convergence speed of the metaheuristic algorithm. When the value of inertia weight is larger, the global search ability of the algorithm is stronger and the search range is wider, which is beneficial for the algorithm to jump out of local optimum and improve the convergence speed. When the value of inertia weights is small, it is beneficial to the algorithm for fine mining in the optimal domain and improving the convergence accuracy of the algorithm. The linear inertia weights proposed by Shi and Eberhart in [30] are often used in metaheuristic algorithms due to their simple structure, ease of use, and better convergence of the algorithm. However, the inertia weights that change linearly with the number of iterations do not accurately reflect the actual optimization process of the GWO algorithm with high nonlinearity, which is not conducive to the global convergence of the algorithm and easily causes the phenomenon of premature algorithm.

Therefore, in this paper, nonlinear inertia weights with adaptive features are introduced in the gray wolf pack hunting process of the GWO algorithm. The performance balance between global search and local mining of GWO algorithm is optimized to accelerate the convergence speed of the algorithm and improve the convergence accuracy, as shown in Equations (12) and (13).

(12) $$\left\{\begin{array}{l} W_{1}={\left(1-\frac{t}{Max_{Iter}}\right)}^{3}\\ W_{2}=\left(\frac{Fit_{t,i}}{Fit_{t,i}+Fit_{t,g}}\right) \end{array}\right.$$

(13) $$W_{GWO}=W_{1}\cdot W_{2}$$

where $W_{GWO}$ denotes the inertia weight of the current gray wolf pack. $Fit_{t,i}$ denotes the fitness value of the ith gray wolf during the tth iteration. $Fit_{t,g}$ denotes the fitness value of the optimal gray wolf during the tth iteration. From Equations (12) and (13), it can be seen that the inertia weight $W_{GWO}$ of the gray wolf pack is expressed as the product of inertia parameters $W_{1}$ and $W_{2}$. The value range of $W_{1}$ is between [0, 1] and will show a decreasing trend of deceleration with the increase in the number of iterations. Larger values of weights in the early iteration of the algorithm facilitate the GWO algorithm to quickly focus on the optimal region and accelerate the global convergence of the algorithm. Smaller weight values in the late iteration of the algorithm are beneficial to the deep mining of the algorithm in the optimal region, improving the convergence accuracy of the GWO algorithm. The inertia parameter $W_{2}$ is related to the fitness of individual gray wolves and is automatically adjusted based on the interpolation between the current individual and the optimal individual. The improved wolf hunting expression of GWO algorithm is shown in Equation (14).

(14) $$\left\{\begin{array}{c} \vec{D_{\alpha }}=|\vec{C_{1}}\cdot \vec{X_{\alpha }}-W_{GWO}\vec{\cdot X}|\\ \vec{D_{\beta }}=|\vec{C_{2}}\cdot \vec{X_{\beta }}-W_{GWO}\cdot \vec{X}|\\ \vec{D_{\delta }}=|\vec{C_{3}}\cdot \vec{X_{\delta }}-W_{GWO}\cdot \vec{X}| \end{array}\right.,\;\left\{\begin{array}{c} \vec{X_{1}}=\vec{X_{\alpha }}-\vec{A_{1}}\cdot (\vec{D_{\alpha }})\\ \vec{X_{2}}=\vec{X_{\beta }}-\vec{A_{2}}\cdot (\vec{D_{\beta }})\\ \vec{X_{3}}=\vec{X_{\delta }}-\vec{A_{3}}\cdot (\vec{D_{\delta }}) \end{array}\right.\;\vec{X}(t+1)=\frac{\vec{X_{1}}+\vec{X_{2}}+\vec{X_{3}}}{3}$$

#### 3.2.3. Random Wandering Law of the Butterfly Optimization Algorithm

The Butterfly Optimization Algorithm (BOA) is a novel metaheuristic algorithm proposed by Sankalap Arora et al., in 2019, which is based on the foraging strategy of butterflies and uses the olfactory sensory organs of butterflies to determine the optimal location of food. Among BOA, scent pheromone is a key determinant of the butterfly’s locomotor state. In the article [31], Sankalap Arora described the relationship between olfactory stimuli and the scent modality of individual butterflies by introducing stimulus intensity (I), perceptual modality (c), and power index (a) as shown in Equation (15).

(15) $$f=cI^{a}$$

where f denotes the intensity at which the scent emitted by the current individual butterfly can be perceived by other butterflies. I denotes the stimulus intensity of the scent produced by the current butterfly, i.e., the current butterfly’s adaptation. c denotes the sensory modality, i.e., simulates the effect of current natural environmental factors on the scent. a denotes the power exponential constant dependent on the scent.

During the iterative calculation of the BOA algorithm, the butterfly’s movement state is influenced by the intensity of its own scent and the intensity of the scent emitted by other individual butterflies. When an individual butterfly senses more scent emitted by another butterfly, it follows and approaches, and this phase is called the global search phase of the BOA algorithm, as shown in Equation (16).

(16) $$x_{i}^{t+1}=x_{i}^{t}+(r^{2}\times g*-x_{i}^{t})\times f_{i}$$

where $x_{i}^{t}$ denotes the ith individual butterfly during the tth iteration. $f_{i}$ denotes the intensity of the scent pheromone emitted by the ith butterfly. g* denotes the fitness that the optimal butterfly individual has in the current iteration. r is denoted as a random number satisfying r∈[0,1]. When a butterfly cannot perceive a scent larger than itself, the individual butterfly will adopt a random wandering strategy. By the influence of other butterfly individuals in the contemporary population, a state of random movement is presented, and this behavior is called the local search of the BOA algorithm, as shown in Equation (17).

(17) $$x_{i}^{t+1}=x_{i}^{t}+(r^{2}\times x_{z}^{t}-x_{k}^{t})\times f_{i}$$

where $x_{z}^{t}$ and $x_{k}^{t}$ denote the zth and kth butterfly individuals of the solution space, respectively.

The GWO algorithm has the disadvantages of higher mining capacity, faster convergence, and smaller memory space occupation in the process of finding the best results [25]. However, the process of iterating the gray wolf population to the optimal area is determined by the positions of Alpha wolves, Beta wolves, and Delta wolves, and then all other wolf packs are guided to follow. The global search by following will lead to a gradual decrease in population diversity and a weakening of the global search capability in the later iterations of the algorithm. Therefore, this paper further improves the local search ability of the GWO algorithm and enhances the population diversity of the algorithm in the late iteration by introducing the scent pheromone and the random wandering law of the BOA algorithm to guide the overall wolf population by random other species of wolves in the pack with a certain probability. In this paper, the key pseudo-code of the random wandering law based on the BOA Algorithm 2:
**Algorithm 2** GWO algorithm based on the randomized wandering law
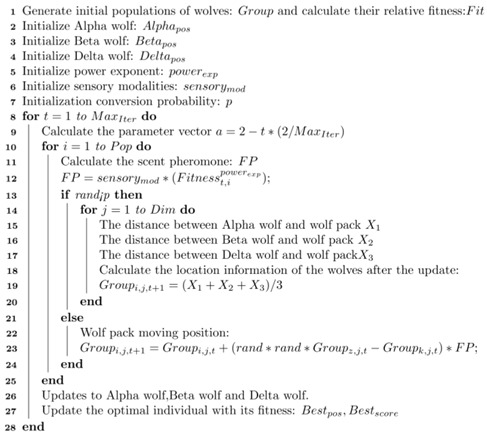


#### 3.2.4. Opposition-Based Learning Method Based on the Elite Strategy

The opposition-based learning method is an intelligent computing approach that relies on the reverse-learning thinking proposed by Tizhoosh in [32]. At present, this method has been widely used in population intelligence algorithms, such as the genetic algorithm [33], particle swarm algorithm [34], artificial bee colony algorithm [35], and differential evolution algorithm [36], to improve the search performance of the algorithm. Qian et al. optimized the initial population generation strategy of the sparrow search algorithm by using a opposition-based learning strategy in [37]. From the experimental results, opposition-based learning has a very good effect on improving the global search efficiency of the population quality enhancement algorithm of the search algorithm.

This paper is based on the integration of elite strategies with opposition-based learning. By selecting elites, the opposite wolf pack is merged with the current pack, and the best individuals are selected to enter the next iteration cycle for searching. That is, while maintaining the diversity of wolf packs to a certain extent and preventing the algorithm from falling into local optimum, it also fully absorbs the beneficial information of the elite individuals in the current wolf packs to accelerate the global convergence speed of the algorithm. Suppose $x_{i}(t)$ and $x_{i}^{*}(t)$ are the current individual and the reverse individual during the tth iteration, respectively, let $Fit_{i,j}(t)$ and $Fit_{i,j}^{*}(t)$ be the adaptation values on the jth dimension corresponding to $x_{i}(t)$ and $x_{i}^{*}(t)$, respectively. In this paper, m(2≤m≤N) elite individuals are represented as shown in Equation (18).

(18) $$\left\{e_{1}(t),e_{2}(t),\ldots ,e_{m}(t)\}\subseteq \{x_{1}(t),x_{2}(t),\ldots ,x_{N}(t)\right\}$$

Then, the reverse solution *F* is defined as shown in Equation (19).

(19) $$Fit_{i,j}^{*}(t)=\eta (a_{j}(t)+b_{j}(t))-Fit_{i,j}(t)\;\left\{\begin{array}{c} a_{j}(t)=\min(e_{1,j}(t),e_{2,j}(t),\ldots ,e_{m,j}(t))\\ b_{j}(t)=\max(e_{1,j}(t),e_{2,j}(t),\ldots ,e_{m,j}(t)) \end{array}\right.$$

where η is a random number and satisfies η∈(0,1).

#### 3.2.5. EWB-GWO Algorithm Flow

In this paper, we combine Tent–Sine two-dimensional chaotic mapping, nonlinear adaptive inertia weights, a random wandering strategy based on the BOA algorithm, and an opposition-based learning method based on elite strategy to improve the shortcomings of the GWO algorithm in processing high-dimensional complex functions with slow convergence speed, low convergence accuracy, and an algorithm prone to falling into a local optimum, and we propose the EWB-GWO algorithm (see Algorithm 3), whose pseudo-code is shown below.
**Algorithm 3** Pseudocode for EWB-GWO algorithm 
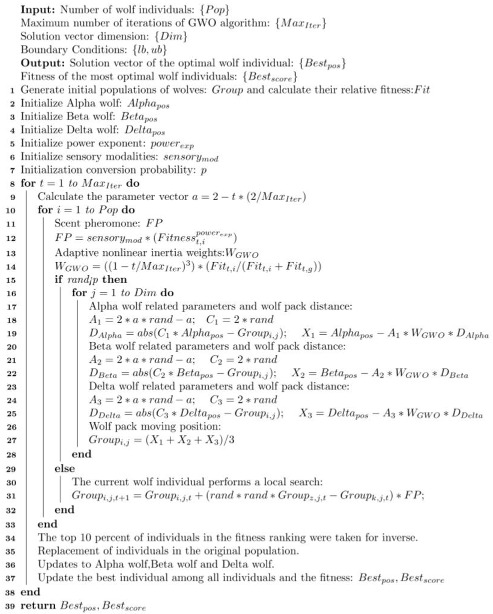


## 4. Path Planning Design Based on EWB-GWO Algorithm

**Figure 6:**
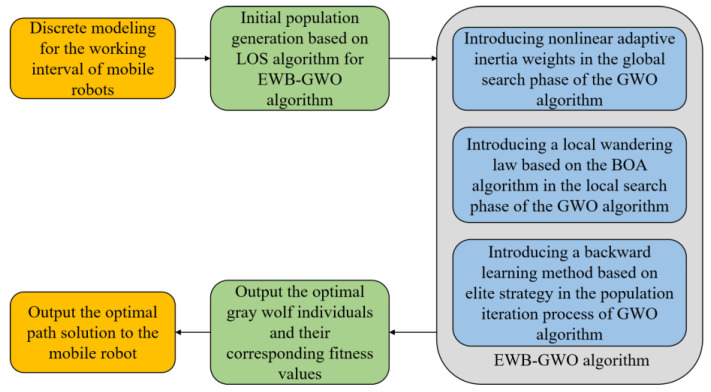
Workflow for mobile robot path planning based on EWB-GWO algorithm.

For the path planning technology of mobile robots performing unmanned delivery tasks, the path length metrics, obstacle avoidance capability, and the operating speed of the algorithm need to be comprehensively considered [38]. The path planning algorithm needs to generate reliable paths under the condition of ensuring the safety and timeliness of the mobile robot. Therefore, this paper will use the EWB-GWO algorithm and combine it with a line-of-sight (LOS)-based initial population strategy to implement an unmanned delivery task based on mobile robots. The flow of the path planning strategy used in this paper is shown in Figure 6 below.

### 4.1. Initial Population Generation Strategy Based on LOS Algorithm

The generation quality of the initial gray wolf population has an important impact on the convergence speed and computational accuracy of the EWB-GWO algorithm in dealing with the path planning problem. The use of random sampling of path interruption points leads to the generation of a large number of infeasible solutions, which reduces the population diversity of the algorithm and increases the computational pressure of the EWB-GWO algorithm.

In this paper, we propose an initial population generation strategy based on LOS detection, which further reduces the number of path nodes while outputting the diversity of feasible solutions and ensures the quality and effectiveness of the initial population. LOS detection is usually performed by the Bresenham’s line algorithm. The Bresenham’s line algorithm is used in computer bitmap inspection, and it is used to detect the individual pixels that pass between two pixel nodes. LOS detection is used in a discrete 2D grid map to determine whether two nodes are directly accessible and free from obstructions. The specific steps of the initial population generation strategy proposed in this paper are shown below.

**Figure 7:**
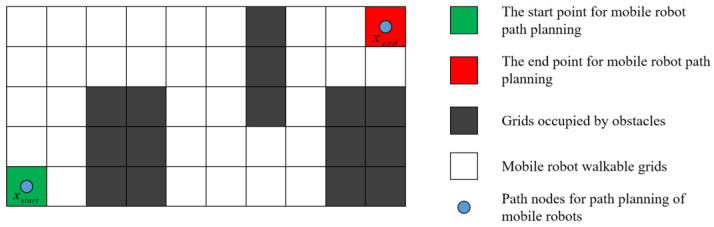
Mobile robot workspace based on grid slice.

**Figure 8:**
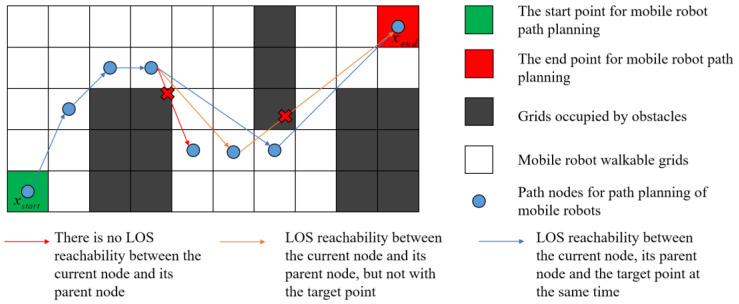
Determine if the current node needs to be retained by LOS detection.

1. The workspace of the mobile robot is modeled as a discrete slice through an equal-area grid. The grid volume is set to 1.2 to 1.5 times the volume of the mobile robot, as shown in Figure 7;
2. The feasible path nodes are generated by Tent–Sine 2D chaotic mapping, and the LOS reachability between all generated nodes and their parents and target nodes is detected by the LOS algorithm;
3. If LOS is reached between the current node and its parent and target nodes simultaneously, the sub-individual is output and the next individual is generated. If the current node has LOS reachable only to the parent node, the current node is kept as the parent node and the path node continues to be generated. If there is no reachability between the current node and the parent node, the current node is directly discarded, as shown in Figure 8;
4. The initial population is generated by iteration until all wolves are viable to end the algorithm.

### 4.2. Mathematical Model Based on EWB-GWO Path Planning

This section is not mandatory but can be added to the manuscript if the discussion is unusually long or complex. We denote the starting point of the mobile robot as $x_{start}$, the target point as $x_{end}$, and the path interruption point as $x_{i}$. Then, the expression of the path corresponding to the gray wolf individual i is shown in Equation (20).

(20) $$\begin{array}{ll} path_{i}= & \left\{x_{start},x_{1},x_{2},x_{3},\ldots ,x_{k},\ldots ,x_{n}|x_{k}\in P_{free}\right\}\\ & n\in [0,\max(length_{x},length_{y})] \end{array}$$

where $P_{free}$ denotes the barrier-free area where the mobile robot can travel. $\max(length_{x},length_{y})$ indicates the maximum number of grids occupied by the *x*-axis and *y*-axis of the working interval of the mobile robot.

During the iterative computation of the EWB-GWO algorithm, the quality of the generated paths is judged by the fitness assessment of the path results represented by the wolf pack individuals through the fitness function. In this paper, the fitness function is established by using the Euclidean distance formula, as shown in Equation (21).

(21) $$\begin{array}{c} Fit(path_{i})=\sqrt{{\left(x_{start.x}-x_{1.x}\right)}^{2}+{\left(x_{start.y}-x_{1.y}\right)}^{2}}+\sum_{k=1}^{n-1}\sqrt{{\left(x_{k+1.x}-x_{k.x}\right)}^{2}+{\left(x_{k+1.y}-x_{k.y}\right)}^{2}}+\\ \sqrt{{\left(x_{n.x}-x_{end.x}\right)}^{2}+{\left(x_{n.y}-x_{end.y}\right)}^{2}} \end{array}$$

**Figure 9:**
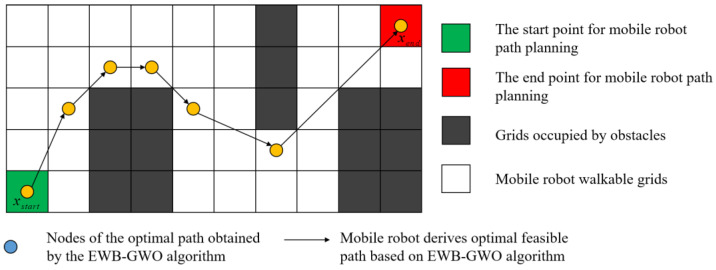
Complete path example for unmanned delivery robot path planning.

The complete search path of the unmanned delivery robot based on the EWB-GWO algorithm is shown in Figure 9.

## 5. Algorithm Simulation and Experimental Design

In the algorithm simulation and experimental design section, 24 CEC benchmark functions designed from different articles are selected for algorithm testing in order to verify the actual operation of the EWB-GWO algorithm proposed in this paper. These benchmark functions can be found in the articles [23,31,38], as shown in Table 1. They contain the name, expression, variable dimension, and theoretical minimum of each function. All experiments in this paper were conducted on an i5-1135G7 minicomputer and using Matlab 2018b software. The experiments as well as the methods used in this paper are described below.

1. In order to verify the improved strategies proposed in this paper, as well as the actual outcome-seeking ability of the improved algorithms, this paper performs benchmark function tests for the GWO algorithm, the EWB-GWO algorithm, and its improved three-seeded strategy algorithm. The improved three sub-strategy algorithms are the GWO algorithm based on the nonlinear adaptive inertia weighting strategy (W-GWO), the GWO algorithm based on the BOA free-roaming strategy (B-GWO), and the GWO algorithm based on the opposition-based learning method of the elite strategy (E-GWO). Three unimodal functions, three multimodal functions, and three multimodal functions with fixed dimensionality are selected for testing in the cases of dimensionality (Dim) 50 and 100 to verify the effectiveness of the improvement strategy proposed in this paper;
2. In this paper, the proposed EWB-GWO algorithm is tested against the extended algorithm that has been optimized and improved for the GWO algorithm in recent years under benchmark functions. In the comparison experiments, the I-GWO algorithm, GWO-CS algorithm, PSO-GWO algorithm, and the original GWO algorithm, which have better support for the benchmark function test, are selected to participate in the tests in order to analyze the superiority of the optimization method of the proposed GWO algorithm over the improved algorithms of the same type;
3. In this paper, four metaheuristic algorithms, namely the PSO algorithm, genetic algorithm (GA), artificial bee colony (ABC) algorithm, and butterfly optimization algorithm (BOA), which are often used to solve complex optimization problems, are selected for cross-sectional comparison experiments with our proposed EWB-GWO algorithm. Additionally, we further analyze the performance of the improved algorithm proposed in this paper compared with the mainstream intelligent optimization algorithms;
4. This paper conducts mobile robot path planning experiments based on the Moving AI Lab public map dataset. This experiment will use different sizes of map data to simulate the real environment. In this experiment, the EWB-GWO algorithm will be compared with the A* and Jump Point Search (JPS) algorithms based on graph search technique, RRT algorithm based on probabilistic sampling, BOA algorithm based on metaheuristic technique, and the Theta* algorithm based on an any-angle path planning strategy.

### 5.1. Benchmark Function Test of EWB-GWO Algorithm and Its Sub-Algorithms

**Figure 10:**
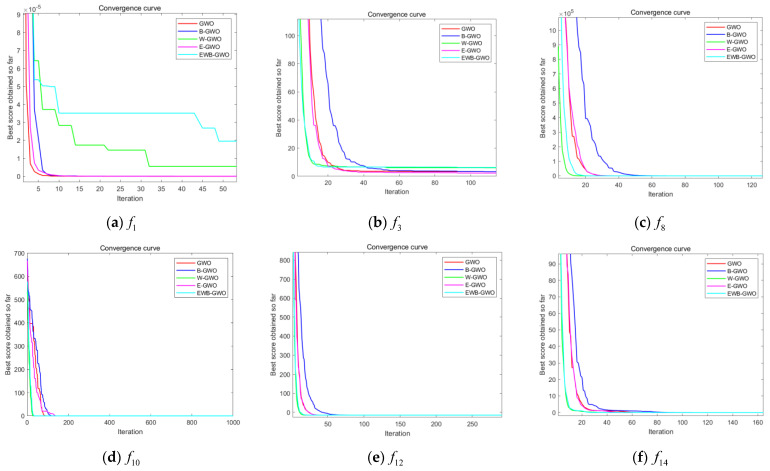
Comparison of convergence curves of GWO, E−GWO, W−GWO, B−GWO, and EWB−GWO with Dim = 50.

**Figure 11:**
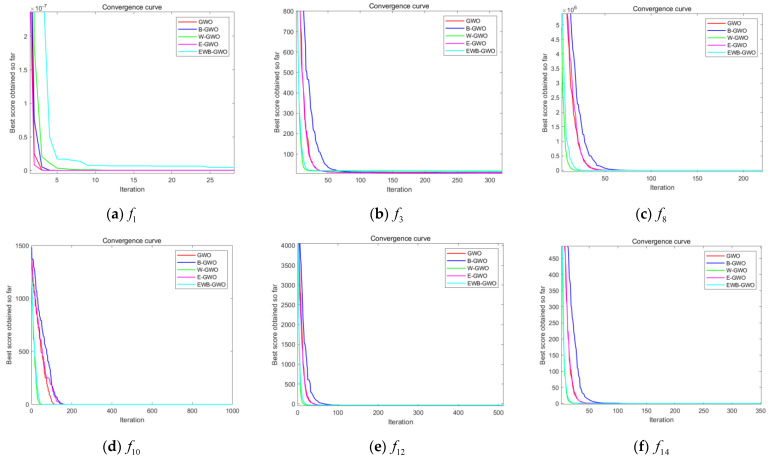
Comparison of convergence curves of GWO, E−GWO, W−GWO, B−GWO, and EWB−GWO with Dim = 100.

**Figure 12:**
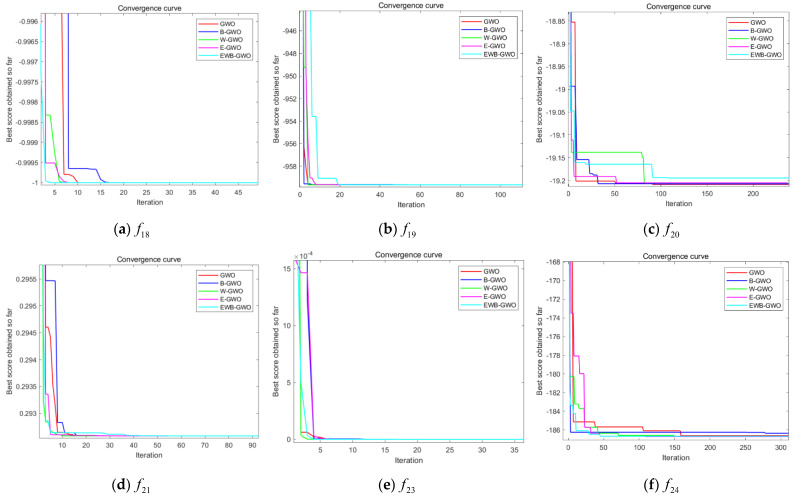
Comparison of convergence curves of GWO, E−GWO, W−GWO, B−GWO, and EWB−GWO in fixed-dimensional multimodal function tests.

In this experiment, three unimodal functions ($f_{1}$,$f_{3}$,$f_{8}$), three multimodal functions ($f_{10}$,$f_{12}$,$f_{14}$), and six fixed-dimension multimodal functions ($f_{18}$,$f_{19}$,$f_{20}$,$f_{21}$,$f_{23}$,$f_{24}$) are randomly selected from Table 1 to compare the GWO algorithm, the EWB-GWO algorithm, and the three improved sub-algorithms in Dim = 50 and Dim = 100 dimensions, respectively. The initial number of wolves in the experimental design is set to 300, and the maximum number of iterations is set to 1000. The experimental results are shown in Figure 10, Figure 11 and Figure 12. In order to better demonstrate the variability in convergence speed between the algorithms, the experimental images are enlarged to different degrees in this paper.

It is clear from the three different sets of convergence tests that the EWB-GWO algorithm as well as the W-GWO algorithm converge the fastest in the tests of $f_{3}$ and $f_{8}$ functions under the single-mode functions, far outperforming the other improved algorithms and the original GWO algorithm, and still leading when the dimensionality rises to Dim = 100. In the test of $f_{1}$ function, the convergence speed of the GWO algorithm and E-GWO algorithm is faster; however, with the increase in dimensionality, the gap of the convergence speed of the algorithm grows gradually smaller from Dim = 50 to Dim = 100, and the shortcomings of the GWO algorithm in dealing with high-dimensional problems are gradually magnified. From the perspective of multimode functions, the advantage of the convergence speed of the EWB-GWO algorithm and W-GWO algorithm is more obvious as the complexity of the benchmark function increases. In the tests of the $f_{10}$, $f_{12}$, and $f_{14}$ functions, the EWB-GWO algorithm and W-GWO algorithm add adaptive nonlinear inertia weights in the process of global search to speed up the convergence of the global search, meaning the algorithm can quickly converge to the global optimum. The EWB-GWO algorithm converges fastest with the W-GWO algorithm under the $f_{18}$, $f_{21}$, and $f_{23}$ functions during the testing of the six fixed-dimensional multimode functions. However, it is undeniable that the EWB-GWO algorithm performs poorly under the $f_{19}$ and $f_{20}$ functions, while there is a small difference in the convergence speed of each algorithm in the $f_{24}$ function test. From the overall experimental results of the 18 groups based on an nonlinear adaptive weighting strategy, to a certain extent there is an acceleration of the global convergence of the algorithm, which means the algorithm can quickly focus on the optimal region for mining, ensuring the search efficiency of the algorithm. In this paper, the optimal (Best), average (Avg), and standard deviation (Std) of the fitness of all the benchmark functions designed in this experiment after 30 runs are shown in Table 2, Table 3 and Table 4, and the search accuracy of the algorithm is further analyzed.

From Table 2 and Table 3, it can be seen that the convergence accuracy is relatively good during the testing of the unimodal functions ($f_{1}$,$f_{3}$,$f_{8}$). Among them, the E-GWO algorithm has the highest convergence accuracy during the testing of the $f_{1}$ function, and the GWO algorithm, B-GWO algorithm, and EWB-GWO algorithm have a more similar convergence accuracy. In the $f_{3}$ and $f_{8}$ function tests, the EWB-GWO algorithm, on the other hand, shows excellent convergence accuracy and is closer to the theoretical optimum than other algorithms in the same dimensional conditions. The E-GWO algorithm and the EWB-GWO algorithm have the best convergence accuracy in the test of the multimodal function ($f_{10}$,$f_{12}$,$f_{14}$), which basically coincides with the theoretical best value. The convergence accuracy of the original GWO algorithm and the B-GWO algorithm is relatively low, and the depth mining ability for the optimal region is insufficient. From Table 4, the difference of the five algorithms for the multimodal functions with fixed dimensions in terms of their ability to find the best result is small and consistent with the theoretical optimum.

From the results of this experiment, it is observed that the nonlinear adaptive inertia weighting strategy proposed in this paper makes the B-GWO algorithm and EWB-GWO algorithm perform well in global convergence speed and far outperform other improved strategies in the tests of unimodal functions, multimodal functions, and multimodal functions with fixed dimensions.

### 5.2. Comparison of Benchmark Functions of Other Improved GWO Algorithms of EWB-GWO Algorithm

**Figure 13:**
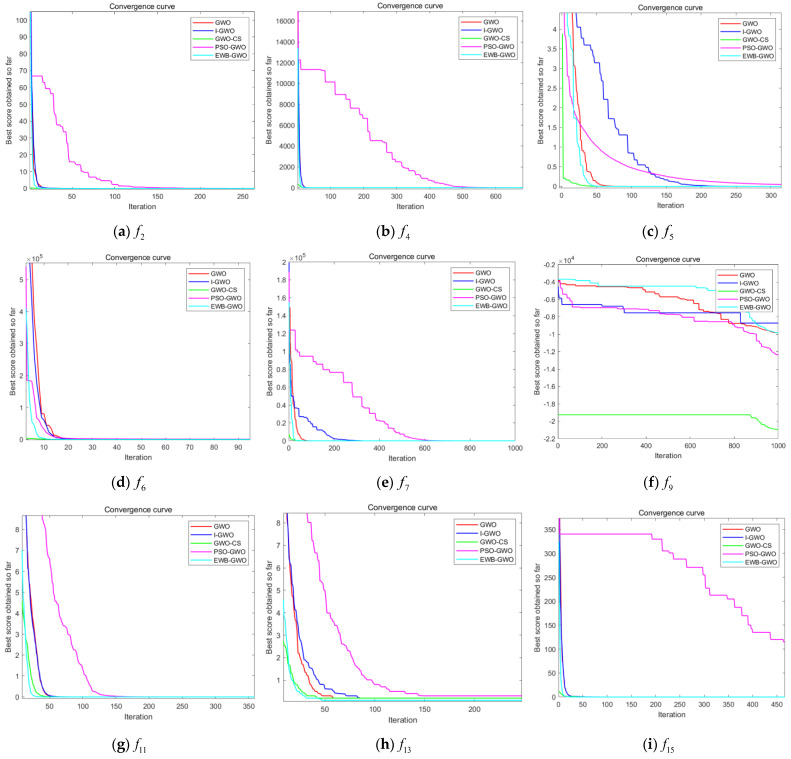

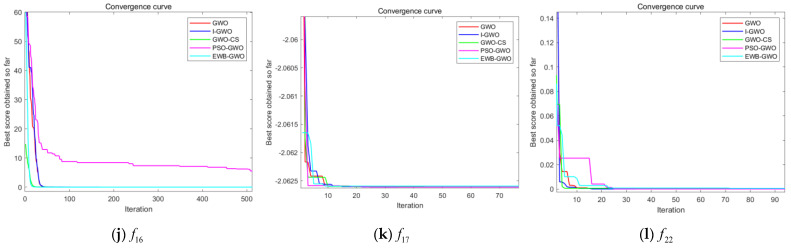
Comparison of convergence curves of GWO, I−GWO, GWO−CS, PSO−GWO, and EWB−GWO.

In this experiment, we will select other benchmark functions from Table 1 that are not involved in the test in Experiment 5.1, which are five unimodal functions ($f_{2}$,$f_{4}$,$f_{5}$,$f_{6}$,$f_{7}$), five multimodal functions ($f_{9}$,$f_{11}$,$f_{13}$,$f_{15}$,$f_{16}$), and two fixed-dimension multimodal functions ($f_{17}$,$f_{22}$) for the cross-sectional comparison verification among GWO-improved algorithms. Since the GWO algorithm performs better with multi-peaked functions of fixed dimensions, only fewer functions are selected for testing and justification in this experiment to avoid a repetition of the conclusions. According to the principle of experimental design fairness, this experiment will use dimension Dim = 50, the number of initial wolf packs is 300, and the maximum number of iterations is 1000. The cross-sectional comparison is verified based on GWO, I-GWO, GWO-CS, PSO-GWO, and EWB-GWO algorithms. The experimental results are shown in Figure 13.

From the convergence curves of each GWO-improved algorithm shown in Figure 13, it can be seen that the GWO-CS algorithm and the EWB-GWO algorithm converge faster compared with the other algorithms. The GWO-CS algorithm converges to the optimum first in the tests of $f_{2}$,$f_{5}$,$f_{6}$,$f_{7}$, and $f_{9}$ functions. The GWO-CS algorithm benefits from the global search method of the Cuckoo Search algorithm by a Lévy flight update of the nest location, which further optimizes the global search performance of the GWO algorithm and further improves the population superiority during the iteration of the algorithm. From the experimental process, it can be found that the initial population quality of the GWO-CS algorithm, as well as the quality of individuals during the population iteration, exceeds other algorithms. The proposed EWB-GWO algorithm converges fastest in the tests of $f_{11}$, $f_{13}$, and $f_{16}$ functions, and the convergence speed of $f_{4}$ and $f_{15}$ functions is basically the same as that of the GWO-CS algorithm. The convergence speed of the PSO-GWO algorithm is the slowest during the experiment, while the convergence speed of the I-GWO algorithm is not much different from the original GWO algorithm; moreover, even the convergence speed is slower than the original GWO algorithm in the $f_{5}$, $f_{7}$, and $f_{9}$ function tests. For the optimization of the global convergence speed, the I-GWO algorithm and the PSO-GWO algorithm are not as effective. In this paper, the convergence results of each algorithm during the experiments are shown in Table 5 and are compared and illustrated in terms of the mean (Avg), standard deviation (Std), optimal value (Best), and running time (Time) under the same parameter settings.

From the experimental results in Table 5, the EWB-GWO algorithm obtains the convergence results closest to the theoretical optimum with the $f_{4}$, $f_{5}$, $f_{7}$, $f_{11}$, $f_{13}$, and $f_{15}$ functions in the comparative experimental tests of the five algorithms, and takes the shortest computation time among the four improved GWO algorithms. The EWB-GWO algorithm has excellent test results in the tests of multi-peak functions as well as multi-peak functions with fixed dimensions. Except for the $f_{9}$ function, all the tested functions basically reach the theoretical optimal value. The convergence accuracy of the PSO-GWO algorithm is more unsatisfactory, and the standard deviation of the function test results shows that the volatility of the PSO-GWO algorithm solution is large, meaning the algorithm is not stable. The EWB-GWO algorithm is highly competitive among GWO algorithms of the same type in terms of convergence speed and convergence accuracy in the test of high-dimensional complex functions.

### 5.3. Comparison of EWB-GWO Algorithm with Other Metaheuristic Algorithms

In this experiment, GWO, B-GWO, W-GWO, E-GWO, EWB-GWO, PSO, GA, BOA, and ABC algorithms are selected for cross-sectional comparison tests based on all the benchmark functions proposed in this paper. Where the unimodal and multimodal functions are performed in the same dimension, i.e., Dim = 50, the test results will be the mean (Avg) and standard deviation (Std) of 30 experiments. In this paper, the parameter design of the cross-sectional comparison algorithm involved in the experiments is summarized in Table 6, and the articles referenced by the parameters are marked.

**Figure 14:**
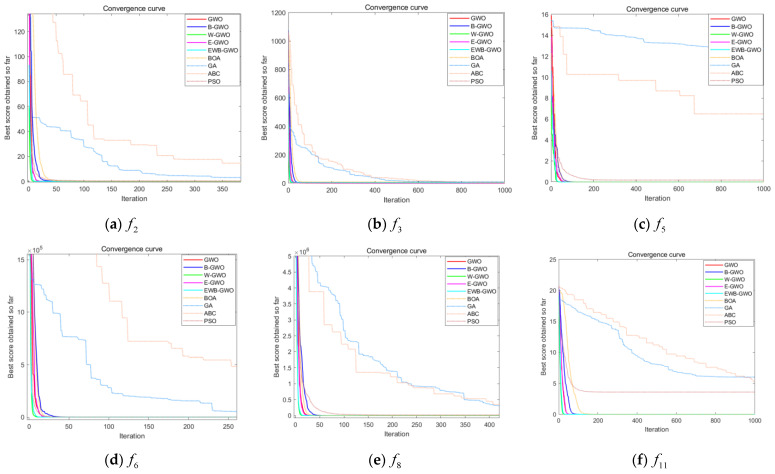

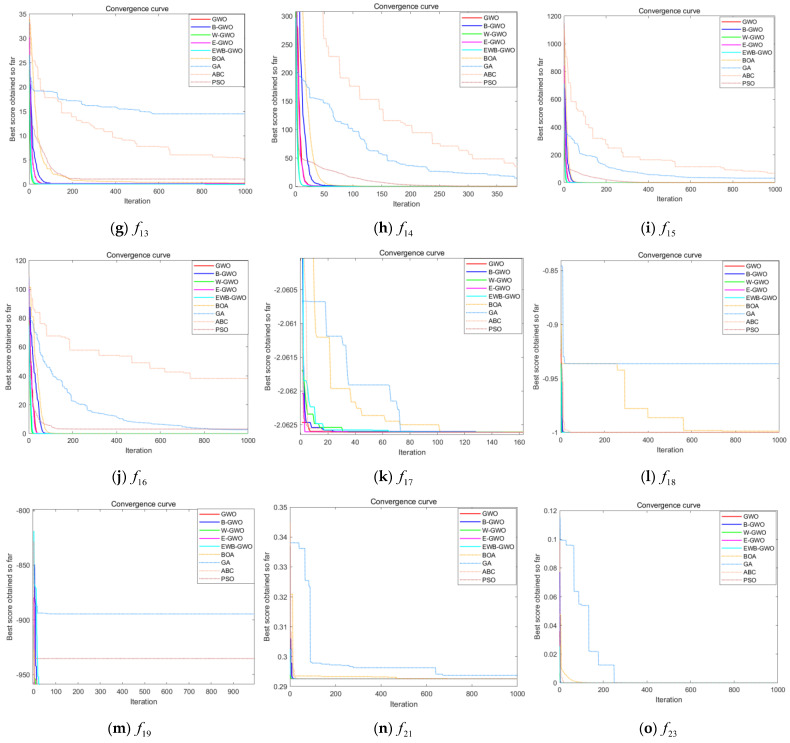
Comparison of convergence curves of GWO, B−GWO, W−GWO, E−GWO, EWB−GWO, GA, PSO, BOA, and ABC.

The parameters about the improved GWO algorithm have been explained in the paper. The convergence curves of some experiments are shown in Figure 14. In order to better demonstrate the variability of convergence speed between the algorithms, the experimental images are enlarged to different degrees in this paper.

It can be seen from the convergence results that the global convergence speed and the ability to jump out of the local optimum of the EWB-GWO algorithm are better compared with other metaheuristics of the same type. Except for $f_{17}$, the EWB-GWO algorithm converges to the theoretical value faster than the other eight algorithms. From the testing process of $f_{5}$, $f_{6}$, $f_{11}$, $f_{13}$, $f_{18}$, and $f_{19}$, it can be found that the GA algorithm and ABC algorithm have different degrees of local optimal stagnation, which makes the algorithm fall into the local solution, becoming unable to escape because the accuracy of the algorithm is low. In particular, the GA algorithm fails to converge in the tests of complex multimodal functions $f_{13}$, $f_{18}$, and $f_{19}$. The convergence of the BOA and PSO algorithms is more stable and can converge quickly to the theoretical optimum during the testing of single-mode functions; however, the convergence speed is still not satisfactory in the testing of multimode complex functions $f_{11}$, $f_{17}$, and $f_{19}$. By analyzing the test of high-dimensional function $f_{14}$, it is not difficult to find that the EWB-GWO algorithm and W-GWO algorithm benefit from the nonlinear adaptive weighting strategy, which enhances the global convergence speed of the algorithm in dealing with high-dimensional complex functions to a certain extent, meaning the algorithm can achieve a fast focus on the optimal interval. B-GWO improves the diversity of the algorithm population to some extent due to the inclusion of the local random wandering law in the iterative process of the algorithm; however, this comes at the expense of the global convergence speed of the algorithm. The convergence results of each algorithm are shown in Table 7.

From the convergence results of each algorithm, the test values of all benchmark functions of the EWB-GWO algorithm, except $f_{7}$, $f_{11}$, $f_{16}$, and $f_{22}$, are close to the theoretical optimum, and their convergence accuracy is much higher than other algorithms. Thanks to the backward-learning method based on the elite strategy, the E-GWO algorithm continuously optimizes the population quality by retaining elites in the process of iterative computation, which further improves the convergence accuracy of the algorithm. The convergence accuracy of the E-GWO algorithm exceeds that of the EWB-GWO algorithm in the test functions $f_{7}$, $f_{11}$, and $f_{16}$, and it also has excellent convergence performance in the remaining functions. The gap between the convergence results of the GA and ABC and the theoretical optimum in the testing process of all high-dimensional functions is large, and the two algorithms exist to different degrees in the testing of high-dimensional functions from $f_{1}$ to $f_{17}$ in the phenomenon of falling into a local optimum that cannot be escaped. In contrast, PSO and BOA show greater volatility in the convergence tests of high-dimensional functions.

The EWB-GWO algorithm converges to the theoretical optimal value in most of the tested functions. The random wandering law of the BOA proposed in this paper enhances the population diversity of the EWB-GWO algorithm in the computational iteration process to some extent. It reduces the possibility of the EWB-GWO algorithm falling into a local optimum. Meanwhile, the opposition-based learning method based on the elite strategy proposed in this paper further improves the quality of elite individuals in the process of population turnover and strengthens the local mining ability of the EWB-GWO algorithm. Finally, the balance of the global exploration and local mining ability of the EWB-GWO algorithm is optimized by the adaptive nonlinear inertia weight strategy. We will rank and order the algorithms designed during the experiment according to the Wilcoxon test and give the tied rank of the algorithms, as shown in Table 8. The *p*-value of Frideman test is also used to further determine the similarity between each metaheuristic algorithm, as shown in Table 9. The analysis method of similarity can be found in article [22].

As can be seen in Table 7, the EWB-GWO algorithm obtained the best rank in 19 out of 24 sets of benchmark function tests, showing the best convergence accuracy. The E-GWO and B-GWO algorithms are the second and third most effective, followed by the W-GWO, GWO, BOA, PSO, ABC, and GA, respectively. As can be seen in Table 8, there are 13 groups of experiments with *p*-values less than 0.05 in the benchmark function test for high-dimensional unimodal and multimodal functions ($f_{1}$~$f_{16}$). In most of the high-dimensional functions tested, there was significant variability among the metaheuristics involved in the cross-sectional comparisons. However, the *p*-value of the multimodal function ($f_{17}$~$f_{24}$) in fixed dimensions shows less variability among the algorithms.

The rank classification and Friedman test supported the results and analysis of this experiment. That is, the improved strategy proposed in this paper makes the EWB-GWO algorithm exhibit excellent global convergence speed and local convergence accuracy in high-dimensional complex functions, which are highly competitive among metaheuristics of the same type.

### 5.4. EWB-GWO Algorithm-Based Path Planning Experiment for Mobile Robot

**Figure 15:**
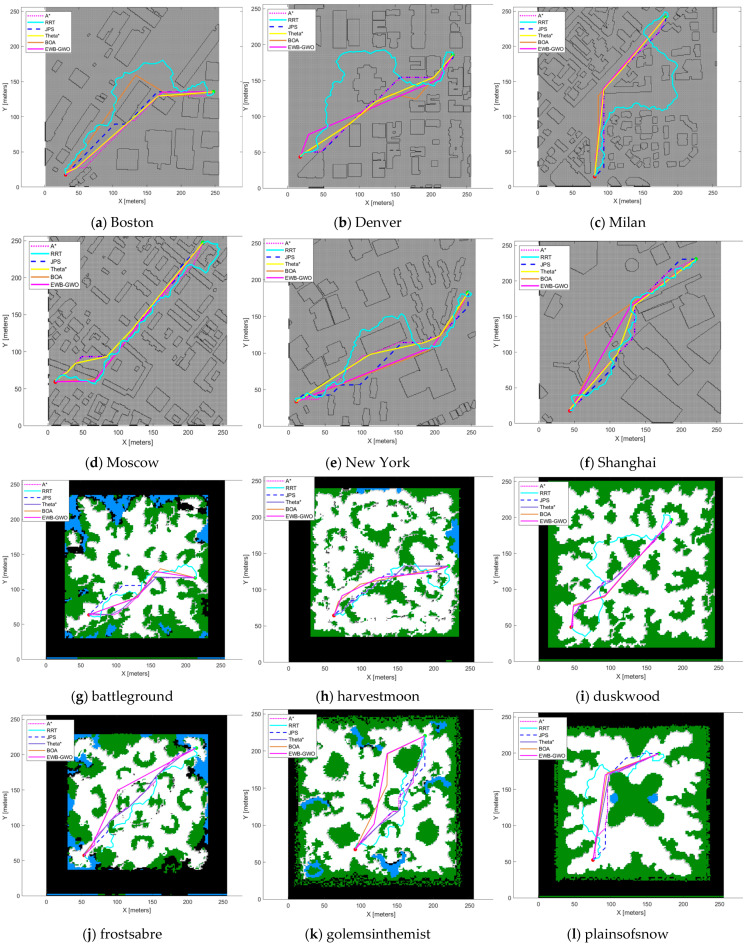
Simulation diagram of drone path planning experiment.

In the experiments of the EWB-GWO algorithm for mobile robot path planning, six game maps (simulating complex field environments) and six city maps from the public map dataset provided by Moving AI Lab (https://movingai.com/, accessed on 10 January 2023) were used for the algorithm comparison tests. In this paper, the information of the selected maps is organized as shown in Table 10. In the process of algorithm comparison, the A* and Jump Point Search (JPS) algorithms based on graph search technique, RRT algorithm based on probabilistic sampling, BOA algorithm based on metaheuristic technique, and Theta* algorithm based on any-angle path planning strategy were selected for this experiment to conduct cross-sectional comparison experiments among different techniques. From the perspective of fairness regarding the experimental design, the heuristic functions of the A*, Theta*, and JPS algorithms used in the path planning experiments adopted the Euclidean distance formula. The step size of the RRT algorithm was set to 1 grid distance length, and the same Bresenham’s line algorithm as the EWB-GWO algorithm was used for collision detection. The experimental results of the path planning are shown and illustrated according to the algorithm’s operation time (time complexity), path length, memory occupation size of the algorithm operation (space complexity), the number of path nodes, and path smoothness. The path smoothness was defined as the sum of the total turning angles of the path. The experimental procedure and results are shown in Figure 15.

The results of the path planning test in Figure 15 show that the six path planning algorithms can accomplish the path finding of the mobile robot in different environments and maps of different complexity. Among them, the length of the paths generated by the A* algorithm based on graph search technique and the JPS algorithm are basically the same, and the routes show similarity or symmetry. Since the JPS algorithm adopts a jump search method to make the number of nodes in the search process drop significantly, the JPS algorithm has an advantage over the A* algorithm in terms of time complexity. Additionally, JPS algorithm has lower memory usage than the A* algorithm due to the decrease in the number of search nodes. However, it is undeniable that the paths generated by the A* and JPS algorithms are only based on the shortest paths under the discrete grid map. Both algorithms are simultaneously limited by the search direction due to the gridization of the workspace in the path finding process, resulting in paths that are not practically optimal. The Theta* algorithm, based on any-angle path planning technology, breaks this limitation. From the experimental process, it can be found that the Theta* algorithm is no longer limited by the search direction, and the obtained paths are closer to the actual optimum. However, the presence of a large number of LOS reachability verifications and angle-value transfer calculations leads to the low operational efficiency of the Theta* algorithm. The RRT algorithm based on probabilistic sampling technique has the advantages of minimal memory usage, simple algorithm structure, and flexible parameter tuning due to its algorithm. However, its path finding by probabilistic sampling leads to an uncontrollable path finding time and generated path quality. In general, the RRT algorithm has a poor quality of generated paths. The BOA algorithm based on the metaheuristic algorithm and the EWB-GWO algorithm both used the initial population generation strategy based on the Bresenham’s line algorithm proposed in this paper during the experiment. From the experimental figure, it can be seen that the path nodes of both algorithms reach the minimum, which substantially reduces the computational difficulty and complexity of the algorithms. At the same time, the two metaheuristics are not limited by the search direction, which makes the metaheuristics have an advantage over graph search techniques and probabilistic sampling techniques in terms of path quality. In this paper, we present the data results of this path planning experiment in Table 11 and Table 12.

In this experiment, path length, path smoothness (total path turning angle), computation time (time complexity), memory consumption (space complexity), and the number of nodes included in the path generated by the algorithm are used as comparison indicators. In terms of path length, the Theta* algorithm is more advantageous than the BOA algorithm and EWB-GWO algorithm based on metaheuristic techniques. The generated path lengths of the three algorithms are relatively similar during most of the experiments. The EWB-GWO algorithm achieves the optimal path length metric in eight sets of experiments, and the Theta* algorithm outperforms the EWB-GWO algorithm in the remaining four sets of experiments. In terms of the path smoothness metric, the metaheuristic algorithm has the same advantage in terms of the total turning angle of the generated paths because it has the lowest number of nodes among the six algorithms. In terms of time complexity and space complexity, the EWB-GWO algorithm, on the other hand, is at a medium level.

From the overall experimental results, the excellent algorithm convergence accuracy and convergence speed possessed by the EWB-GWO algorithm make it more advantageous in terms of path length and total path turning angle. It is undeniable that the EWB-GWO algorithm requires a higher running time than the JPS algorithm; however, the time complexity and space complexity of the EWB-GWO algorithm are lower than the Theta* algorithm, with better generated path quality.

## 6. Conclusions

For mobile robots performing unmanned delivery tasks, the autonomous navigation of the mobile robot will be a decisive factor in determining the success of the delivery task. Path planning technology based on the autonomous decision making of mobile robots has received a lot of attention as a key technology for robot navigation. Under the condition of ensuring the safety of mobile robot operation, a shorter and smoother path length will significantly reduce the robot execution time and improve distribution efficiency. Based on the above objectives, this paper implements a path planning design for unmanned delivery robots through an improved GWO algorithm to accomplish delivery tasks in complex urban and suburban environments while ensuring the safety of mobile robots. From the results of the path planning experiments, the stability of the path solutions generated by the EWB-GWO algorithm is not high, and the time complexity and space complexity of the algorithm need to be further reduced. In future work, we will investigate the situation of dynamic obstacles in the workspace of mobile robots, improving the autonomous decision making of mobile robots.

## Figures and Tables

**Table 1:** The benchmark functions used in this article.

No.	Name	Formula	Dim	Range	$f_{\min}$
Unimodal benchmark functions.					
$f_{1}$	Exponential	$f(x)=\exp(0.5\sum_{i=1}^{n}x_{i})$	50	[−10, 10]	0
$f_{2}$	Quartic	$f(x)=\sum_{i=1}^{n}ix_{i}^{4}+rand(0,1)$	50	[−1.28, 1.28]	0
$f_{3}$	Step	$f(x)=\sum_{i=1}^{n}{\left(\left\lfloor x_{i}+0.5\right\rfloor \right)}^{2}$	50	[−10, 10]	0
$f_{4}$	Sum square	$f(x)=\sum_{i=1}^{n}ix_{i}^{2}$	50	[−10, 10]	0
$f_{5}$	Zakharov	$f(x)=\sum_{i=1}^{n}x_{i}^{2}+{\left(\sum_{i=1}^{n}0.5ix_{i}\right)}^{2}+{\left(\sum_{i=1}^{n}0.5ix_{i}\right)}^{4}$	50	[−1, 1]	0
$f_{6}$	Rosenbrock	$f(x)=\sum_{i=1}^{n-1}\left[100{\left(x_{i+1}-x_{i}^{2}\right)}^{2}+{\left(x_{i}-1\right)}^{2}\right]$	50	[−10, 10]	0
$f_{7}$	Schwefel 1.2	$f(x)=\sum_{i=1}^{n}{\left(\sum_{j=1}^{i}x_{j}\right)}^{2}$	50	[−100, 100]	0
$f_{8}$	Cigar	$f(x)=x_{1}^{2}+10^{6}\sum_{i=2}^{n}x_{i}^{2}$	50	[−1, 1]	0
Multimodal benchmark functions					
$f_{9}$	Schwefel 2.26	$f(x)=-\sum_{i=1}^{n}\left[x_{i}\sin(\sqrt{\left|x_{i}\right|})\right]$	50	[−500, 500]	−12,569.5
$f_{10}$	Rastrigin	$f(x)=\sum_{i=1}^{n}\left(x_{i}^{2}-10\cos(2\pi x_{i})+10\right)$	50	[−5.12, 5.12]	0
$f_{11}$	Ackley	$\begin{array}{l} f(x)=-20\exp\left(-0.2\sqrt{\frac{1}{n}\sum_{i=1}^{n}x_{i}^{2}}\right)\\ -\exp\left(\frac{1}{n}\sum_{i=1}^{n}\cos(2\pi x_{i})\right)+20+\exp(1) \end{array}$	50	[−32, 32]	0
$f_{12}$	Bohachevsky	$f(x)=\sum_{i=1}^{n-1}\left[x_{i}^{2}+2x_{i+1}^{2}-0.3\cos(3\pi x_{i})\right]$	50	[−10, 10]	0
$f_{13}$	Solomon	$f(x)=1-\cos\left(2\pi \sqrt{\sum_{i=1}^{n}x_{i}^{2}}\right)+0.1\sqrt{\sum_{i=1}^{n}x_{i}^{2}}$	50	[−100, 100]	0
$f_{14}$	Griewank	$f(x)=\frac{1}{4000}\sum_{i=1}^{n}x_{i}^{2}-\prod_{i=1}^{30}\cos(\frac{x_{i}}{\sqrt{i}})+1$	50	[−500, 500]	0
$f_{15}$	NCRastrigin	$f(x)=\sum_{i=1}^{n}\left[y_{i}^{2}-10\cos(2\pi y_{i})+10\right]$	50	[−5.12, 5.12]	0
		$y_{i}=\left\{\begin{array}{ll} x_{i} & \left|x_{i}\right|<0.5\\ round(2x_{i})/2 & \left|x_{i}\right|>0.5 \end{array}\right.$			
$f_{16}$	Alpine	$f(x)=\sum_{i=1}^{n}\left|x_{i}\sin(x_{i})+0.1x_{i}\right|$	50	[−10, 10]	0
Fixed-dimension multimodal benchmark functions					
$f_{17}$	Cross-in-Tray	$f(x)=-0.0001{\left(|\sin(x_{1})\sin(x_{2})\exp(|100-\frac{\sqrt{x_{1}^{2}+x_{2}^{2}}}{\pi }|)|+1\right)}^{0.1}$	2	[−10, 10]	−2.06261
$f_{18}$	Drop-Wave	$f(x)=-\frac{1+\cos(12\sqrt{x_{1}^{2}+x_{2}^{2}})}{0.5(x_{1}^{2}+x_{2}^{2})+2}$	2	[−5.12, 5.12]	−1
$f_{19}$	Eggholder	$f(x)=-(x_{2}+47)\sin(\sqrt{\left|x_{2}+\frac{x_{1}}{2}+47\right|})-x_{1}\sin(\sqrt{\left|x_{1}-(x_{2}+47)\right|})$	2	[−512, 512]	−959.641
$f_{20}$	Holder Table	$f(x)=-|\sin(x_{1})\cos(x_{2})\exp(|1-\frac{\sqrt{x_{1}^{2}+x_{2}^{2}}}{\pi }|)|$	2	[−10, 10]	−19.2085
$f_{21}$	Schaffer N.4	$f(x)=0.5+\frac{\cos^{2}(\sin(|x_{1}^{2}-x_{2}^{2}|))-0.5}{{\left[1+0.001(x_{1}^{2}+x_{2}^{2})\right]}^{2}}$	2	[−100, 100]	0
$f_{22}$	Levy N.13	$\begin{array}{c} f(x)=\sin^{2}(3\pi x_{1})+{\left(x_{1}-1\right)}^{2}[1+\sin^{2}(3\pi x_{2})]\\ +{\left(x_{2}-1\right)}^{2}[1+\sin^{2}(2\pi x_{2})] \end{array}$	2	[−10, 10]	0
$f_{23}$	Schaffer N.2	$f(x)=0.5+\frac{\sin^{2}(x_{1}^{2}-x_{2}^{2})-0.5}{{\left[1+0.001(x_{1}^{2}+x_{2}^{2})\right]}^{2}}$	2	[−100, 100]	0
$f_{24}$	Shubert	$f(x)=(\sum_{i=1}^{5}i\cos((i+1)x_{1}+i))(\sum_{i=1}^{5}i\cos((i+1)x_{2}+i))$	2	[−10, 10]	−186.731

**Table 2:** Test results of the algorithm at Dim = 50.

Functions		GWO	B-GWO	W-GWO	E-GWO	EWB-GWO
$f_{1}$	Avg	6.955 × 10^−100^	1.934 × 10^−102^	1.733 × 10^−77^	1.491 × 10^−105^	3.087 × 10^−102^
	Std	1.237 × 10^−99^	2.802 × 10^−102^	3.466 × 10^−77^	2.955 × 10^−105^	6.157 × 10^−102^
	Best	3.302 × 10^−109^	1.766 × 10^−105^	1.732 × 10^−94^	3.153 × 10^−109^	5.015 × 10^−107^
$f_{3}$	Avg	6.102 × 10^−01^	8.754 × 10^−01^	3.101 × 10^00^	9.996 × 10^−02^	9.806 × 10^−02^
	Std	3.719 × 10^−01^	3.710 × 10^−01^	5.146 × 10^−01^	1.999 × 10^−01^	1.959 × 10^−01^
	Best	2.499 × 10^−01^	4.970 × 10^−01^	2.250 × 10^00^	6.503 × 10^−06^	4.672 × 10^−06^
$f_{8}$	Avg	2.079 × 10^−76^	2.287 × 10^−79^	8.434 × 10^−76^	1.325 × 10^−118^	2.514 × 10^−139^
	Std	1.302 × 10^−76^	4.574 × 10^−79^	1.246 × 10^−75^	2.650 × 10^−118^	3.219 × 10^−139^
	Best	4.806 × 10^−77^	6.646 × 10^−88^	3.605 × 10^−77^	6.180 × 10^−140^	1.241 × 10^−148^
$f_{10}$	Avg	0.000 × 10^00^	0.000 × 10^00^	0.000 × 10^00^	0.000 × 10^00^	0.000 × 10^00^
	Std	0.000 × 10^00^	0.000 × 10^00^	0.000 × 10^00^	0.000 × 10^00^	0.000 × 10^00^
	Best	0.000 × 10^00^	0.000 × 10^00^	0.000 × 10^00^	0.000 × 10^00^	0.000 × 10^00^
$f_{12}$	Avg	9.611 × 10^−18^	6.771 × 10^−76^	1.325 × 10^−128^	2.521 × 10^−139^	9.621 × 10^−140^
	Std	1.922 × 10^−17^	9.590 × 10^−76^	2.650 × 10^−128^	3.224 × 10^−139^	1.920 × 10^−139^
	Best	2.007 × 10^−77^	3.605 × 10^−77^	6.180 × 10^−140^	1.415 × 10^−147^	1.325 × 10^−147^
$f_{14}$	Avg	0.000 × 10^00^	0.000 × 10^00^	0.000 × 10^00^	0.000 × 10^00^	0.000 × 10^00^
	Std	0.000 × 10^00^	0.000 × 10^00^	0.000 × 10^00^	0.000 × 10^00^	0.000 × 10^00^
	Best	0.000 × 10^00^	0.000 × 10^00^	0.000 × 10^00^	0.000 × 10^00^	0.000 × 10^00^

**Table 3:** Test results of the algorithm at Dim = 100.

Functions		GWO	B-GWO	W-GWO	E-GWO	EWB-GWO
$f_{1}$	Avg	1.587 × 10^−188^	1.052 × 10^−185^	1.077 × 10^−119^	9.641 × 10^−191^	4.069 × 10^−181^
	Std	0.000 × 10^00^	0.000 × 10^00^	2.153 × 10^−119^	0.000 × 10^00^	0.000 × 10^00^
	Best	3.559 × 10^−196^	1.925 × 10^−203^	1.550 × 10^−143^	4.826 × 10^−199^	2.116 × 10^−194^
$f_{3}$	Avg	4.473 × 10^00^	5.816 × 10^00^	1.167 × 10^01^	4.388 × 10^00^	1.564 × 10^00^
	Std	2.294 × 10^−01^	9.534 × 10^−01^	7.229 × 10^−01^	6.501 × 10^−01^	2.234 × 10^−01^
	Best	4.204 × 10^00^	4.735 × 10^00^	1.075 × 10^01^	3.720 × 10^00^	1.231 × 10^00^
$f_{8}$	Avg	1.514 × 10^−48^	7.696 × 10^−37^	3.172 × 10^−38^	2.528 × 10^−48^	7.847 × 10^−86^
	Std	1.454 × 10^−48^	1.366 × 10^−37^	6.343 × 10^−38^	3.170 × 10^−48^	1.569 × 10^−85^
	Best	3.418 × 10^−49^	5.485 × 10^−37^	3.470 × 10^−98^	2.607 × 10^−49^	5.068 × 10^−99^
$f_{10}$	Avg	6.422 × 10^−13^	0.000 × 10^00^	0.000 × 10^00^	7.276 × 10^−14^	0.000 × 10^00^
	Std	9.060 × 10^−13^	0.000 × 10^00^	0.000 × 10^00^	8.794 × 10^−14^	0.000 × 10^00^
	Best	0.000 × 10^00^	0.000 × 10^00^	0.000 × 10^00^	0.000 × 10^00^	0.000 × 10^00^
$f_{12}$	Avg	9.051 × 10^−40^	5.933 × 10^−27^	3.172 × 10^−38^	2.931 × 10^−47^	7.891 × 10^−86^
	Std	1.810 × 10^−39^	3.291 × 10^−27^	6.343 × 10^−38^	5.340 × 10^−47^	1.578 × 10^−85^
	Best	3.418 × 10^−49^	8.430 × 10^−37^	3.470 × 10^−38^	2.607 × 10^−49^	5.068 × 10^−99^
$f_{14}$	Avg	0.000 × 10^00^	0.000 × 10^00^	0.000 × 10^00^	0.000 × 10^00^	0.000 × 10^00^
	Std	0.000 × 10^00^	0.000 × 10^00^	0.000 × 10^00^	0.000 × 10^00^	0.000 × 10^00^
	Best	0.000 × 10^00^	0.000 × 10^00^	0.000 × 10^00^	0.000 × 10^00^	0.000 × 10^00^

**Table 4:** Test results of the algorithm in fixed-dimensional multimodal function tests.

Functions		GWO	B-GWO	W-GWO	E-GWO	EWB-GWO
$f_{18}$	Avg	−1.000 × 10^00^	−1.000 × 10^00^	−1.000 × 10^00^	−1.000 × 10^00^	−1.000 × 10^00^
	Std	0.000 × 10^00^	0.000 × 10^00^	0.000 × 10^00^	0.000 × 10^00^	0.000 × 10^00^
	Best	−1.000 × 10^00^	−1.000 × 10^00^	−1.000 × 10^00^	−1.000 × 10^00^	−1.000 × 10^00^
$f_{19}$	Avg	−9.596 × 10^02^	−9.596 × 10^02^	−9.596 × 10^02^	−9.596 × 10^02^	−9.596 × 10^02^
	Std	0.000 × 10^+00^	0.000 × 10^00^	0.000 × 10^00^	0.000 × 10^00^	0.000 × 10^00^
	Best	−9.596 × 10^02^	−9.596 × 10^02^	−9.596 × 10^02^	−9.596 × 10^02^	−9.596 × 10^02^
$f_{20}$	Avg	−1.921 × 10^01^	−1.921 × 10^01^	−1.921 × 10^01^	−1.921 × 10^01^	−1.921 × 10^01^
	Std	0.000 × 10^00^	0.000 × 10^00^	0.000 × 10^00^	0.000 × 10^00^	0.000 × 10^00^
	Best	−1.921 × 10^01^	−1.921 × 10^01^	−1.921 × 10^01^	−1.921 × 10^01^	−1.921 × 10^01^
$f_{21}$	Avg	2.926 × 10^−01^	2.926 × 10^−01^	2.926 × 10^−01^	2.926 × 10^−01^	2.926 × 10^−01^
	Std	0.000 × 10^00^	0.000 × 10^00^	0.000 × 10^00^	0.000 × 10^00^	0.000 × 10^00^
	Best	2.926 × 10^−01^	2.926 × 10^−01^	2.926 × 10^−01^	2.926 × 10^−01^	2.926 × 10^−01^
$f_{23}$	Avg	0.000 × 10^00^	0.000 × 10^00^	0.000 × 10^00^	0.000 × 10^00^	0.000 × 10^00^
	Std	0.000 × 10^00^	0.000 × 10^00^	0.000 × 10^00^	0.000 × 10^00^	0.000 × 10^00^
	Best	0.000 × 10^00^	0.000 × 10^00^	0.000 × 10^00^	0.000 × 10^00^	0.000 × 10^00^
$f_{24}$	Avg	−1.867 × 10^02^	−1.867 × 10^02^	−1.867 × 10^02^	−1.867 × 10^02^	−1.867 × 10^02^
	Std	0.000 × 10^00^	0.000 × 10^00^	0.000 × 10^00^	0.000 × 10^00^	0.000 × 10^00^
	Best	−1.867 × 10^02^	−1.867 × 10^02^	−1.867 × 10^02^	−1.867 × 10^02^	−1.867 × 10^02^

**Table 5:** Test results of the GWO-improved algorithms.

Functions		GWO	I-GWO	GWO-CS	PSO-GWO	EWB-GWO
$f_{2}$	Avg	1.057 × 10^−05^	2.447 × 10^−06^	7.562 × 10^−06^	2.088 × 10^−05^	2.981 × 10^−06^
	Std	3.851 × 10^−06^	9.850 × 10^−07^	1.935 × 10^−06^	2.072 × 10^−05^	5.594 × 10^−07^
	Best	7.139 × 10^−06^	1.225 × 10^−06^	4.964 × 10^−06^	3.222 × 10^−06^	2.306 × 10^−06^
	Time	3.352 × 10^−01^	7.030 × 10^+00^	6.236 × 10^+00^	6.470 × 10^+00^	1.973 × 10^+00^
$f_{4}$	Avg	2.073 × 10^−78^	8.802 × 10^−79^	1.070 × 10^−80^	2.408 × 10^−01^	8.261 × 10^−121^
	Std	2.436 × 10^−78^	9.312 × 10^−79^	5.351 × 10^−81^	3.405 × 10^−01^	5.791 × 10^−121^
	Best	2.048 × 10^−79^	2.478 × 10^−80^	5.074 × 10^−81^	2.124 × 10^−44^	2.106 × 10^−122^
	Time	2.110 × 10^+00^	1.097 × 10^+01^	1.192 × 10^+01^	1.068 × 10^+01^	3.616 × 10^+00^
$f_{5}$	Avg	4.329 × 10^−39^	2.338 × 10^−32^	5.938 × 10^−41^	2.284 × 10^+00^	1.754 × 10^−50^
	Std	4.904 × 10^−39^	1.216 × 10^−32^	5.987 × 10^−41^	3.211 × 10^+00^	8.684 × 10^−51^
	Best	2.380 × 10^−40^	1.098 × 10^−32^	6.222 × 10^−42^	4.707 × 10^−26^	7.574 × 10^−51^
	Time	2.305 × 10^+00^	1.178 × 10^+01^	1.186 × 10^+01^	1.284 × 10^+01^	3.809 × 10^+00^
$f_{6}$	Avg	4.546 × 10^−01^	4.024 × 10^−01^	3.164 × 10^−01^	4.819 × 10^−01^	1.648 × 10^+00^
	Std	1.113 × 10^−02^	2.577 × 10^−03^	2.705 × 10^−01^	3.388 × 10^−02^	1.720 × 10^+00^
	Best	4.426 × 10^−01^	3.991 × 10^−01^	1.223 × 10^−01^	4.473 × 10^−01^	4.023 × 10^−01^
	Time	2.179 × 10^+00^	1.126 × 10^+01^	1.216 × 10^+01^	1.173 × 10^+01^	3.746 × 10^+00^
$f_{7}$	Avg	1.257 × 10^−22^	5.937 × 10^−13^	9.554 × 10^−24^	5.874 × 10^−03^	7.805 × 10^−42^
	Std	1.581 × 10^−22^	6.696 × 10^−13^	7.650 × 10^−24^	6.662 × 10^−03^	5.491 × 10^−42^
	Best	8.025 × 10^−24^	5.653 × 10^−15^	1.167 × 10^−24^	1.080 × 10^−15^	8.996 × 10^−43^
	Time	5.552 × 10^+00^	1.846 × 10^+01^	1.524 × 10^+01^	1.595 × 10^+01^	1.348 × 10^+01^
$f_{9}$	Avg	−1.016 × 10^+04^	−1.244 × 10^+04^	−2.095 × 10^+04^	−1.267 × 10^+04^	−1.129 × 10^+04^
	Std	1.178 × 10^+03^	3.257 × 10^+03^	1.843 × 10^−01^	1.295 × 10^+03^	1.849 × 10^+03^
	Best	−1.167 × 10^+04^	−1.534 × 10^+04^	−2.095 × 10^+04^	−1.385 × 10^+04^	−1.267 × 10^+04^
	Time	2.585 × 10^+00^	1.352 × 10^+01^	1.347 × 10^+01^	1.345 × 10^+01^	5.322 × 10^+00^
$f_{11}$	Avg	1.510 × 10^−14^	1.391 × 10^−14^	1.510 × 10^−14^	6.163 × 10^+00^	5.994 × 10^−15^
	Std	0.000 × 10^+00^	1.675 × 10^−15^	0.000 × 10^+00^	8.705 × 10^+00^	2.828 × 10^−15^
	Best	1.510 × 10^−14^	1.155 × 10^−14^	1.510 × 10^−14^	5.773 × 10^−14^	1.994 × 10^−15^
	Time	2.293 × 10^+00^	1.170 × 10^+01^	1.243 × 10^+01^	1.317 × 10^+01^	3.908 × 10^+00^
$f_{13}$	Avg	1.999 × 10^−01^	1.999 × 10^−01^	1.999 × 10^−01^	3.952 × 10^−01^	2.000 × 10^−07^
	Std	0.000 × 10^+00^	0.000 × 10^+00^	0.000 × 10^+00^	8.146 × 10^−02^	2.087 × 10^−07^
	Best	1.999 × 10^−01^	1.999 × 10^−01^	1.999 × 10^−01^	2.938 × 10^−01^	1.348 × 10^−08^
	Time	2.216 × 10^+00^	1.163 × 10^+01^	1.216 × 10^+01^	1.278 × 10^+01^	3.822 × 10^+00^
$f_{15}$	Avg	0.000 × 10^+00^	0.000 × 10^+00^	0.000 × 10^+00^	2.185 × 10^+01^	0.000 × 10^+00^
	Std	0.000 × 10^+00^	0.000 × 10^+00^	0.000 × 10^+00^	3.090 × 10^+01^	0.000 × 10^+00^
	Best	0.000 × 10^+00^	0.000 × 10^+00^	0.000 × 10^+00^	2.220 × 10^−16^	0.000 × 10^+00^
	Time	2.603 × 10^+00^	1.212 × 10^+01^	1.247 × 10^+01^	1.328 × 10^+01^	5.649 × 10^+00^
$f_{16}$	Avg	2.156 × 10^−24^	4.105 × 10^−05^	1.631 × 10^−45^	3.616 × 10^+00^	1.902 × 10^−69^
	Std	3.050 × 10^−24^	3.767 × 10^−05^	1.690 × 10^−45^	1.380 × 10^+00^	1.318 × 10^−69^
	Best	4.470 × 10^−40^	2.328 × 10^−06^	1.169 × 10^−46^	1.856 × 10^+00^	4.990 × 10^−70^
	Time	2.384 × 10^+00^	1.129 × 10^+01^	1.216 × 10^+01^	1.299 × 10^+01^	3.915 × 10^+00^
$f_{17}$	Avg	−2.063 × 10^+00^	−2.063 × 10^+00^	−2.063 × 10^+00^	−2.063 × 10^+00^	−2.063 × 10^+00^
	Std	0.000 × 10^+00^	0.000 × 10^+00^	0.000 × 10^+00^	0.000 × 10^+00^	0.000 × 10^+00^
	Best	−2.063 × 10^+00^	−2.063 × 10^+00^	−2.063 × 10^+00^	−2.063 × 10^+00^	−2.063 × 10^+00^
	Time	2.001 × 10^−01^	6.308 × 10^+00^	2.925 × 10^+00^	5.237 × 10^+00^	2.597 × 10^+00^
$f_{22}$	Avg	9.201 × 10^−10^	1.350 × 10^−31^	9.208 × 10^−09^	4.439 × 10^−09^	3.475 × 10^−08^
	Std	4.977 × 10^−10^	0.000 × 10^+00^	7.000 × 10^−09^	5.726 × 10^−09^	4.496 × 10^−08^
	Best	2.166 × 10^−10^	1.350 × 10^−31^	1.092 × 10^−09^	1.873 × 10^−11^	1.091 × 10^−12^
	Time	1.786 × 10^−01^	6.120 × 10^+00^	2.819 × 10^+00^	3.247 × 10^−01^	2.434 × 10^+00^

**Table 6:** The parameter setting of each algorithm.

Name	Source	Parameter
EWB-GWO	[23]	a∈[0,2]; c=0.01; a=0.1; p=0.8. Elite individuals select 10 percent of the total population.
PSO	[39]	Learning factor of PSO algorithm $c_{1}=1.5$, $c_{2}=1.5$; the maximum value of inertia weight $\omega_{Max}=0.8$; the minimum value of inertia weight $\omega_{Min}=0.7$.
BOA	[31]	Perceptual modality c=0.01; power index a=0.1; conversion probability p=0.8.
ABC	[40]	$T_{limit}=SN\times D$, where $T_{limit}$ denotes the maximum number of update-free iterations of the ABC algorithm for detecting bee solutions, SN denotes the number of food sources, and D denotes the problem dimension.
GA	[41]	Crossover probability $p_{c}=0.9$; variance probability $p_{m}=0.1$.

**Table 7:** BOA-SA, BOA-AIW, BOA-RV, BOA-TSAR, GA, PSO, GWO, and ABC test results on the 50 dimensional functions in Experiment 2.

Functions		GWO	B-GWO	W-GWO	E-GWO	EWB-GWO	BOA	PSO	ABC	GA
$f_{1}$	Avg	1.35 × 10^−104^	6.46 × 10^−103^	8.26 × 10^−84^	3.40 × 10^−107^	5.66 × 10^−107^	1.33 × 10^−28^	5.61 × 10^−73^	1.79 × 10^−101^	5.61 × 10^−73^
	Std	1.33 × 10^−104^	5.12 × 10^−103^	1.17 × 10^−83^	2.39 × 10^−107^	7.06 × 10^−107^	1.88 × 10^−28^	7.94 × 10^−73^	1.25 × 10^−101^	7.93 × 10^−73^
$f_{2}$	Avg	2.14 × 10^−04^	1.60 × 10^−05^	2.02 × 10^−05^	1.73 × 10^−05^	2.90 × 10^−06^	3.07 × 10^−04^	3.64 × 10^−01^	2.01 × 10^00^	1.38 × 10^00^
	Std	9.98 × 10^−05^	1.26 × 10^−05^	2.99 × 10^−06^	1.44 × 10^−05^	1.73 × 10^−06^	8.00 × 10^−05^	6.58 × 10^−02^	4.48 × 10^−01^	2.47 × 10^−01^
$f_{3}$	Avg	6.38 × 10^−01^	1.00 × 10^00^	3.81 × 10^00^	7.03 × 10^−01^	3.98 × 10^−01^	9.18 × 10^00^	1.22 × 10^00^	3.43 × 10^00^	1.51 × 10^01^
	Std	1.61 × 10^−01^	4.11 × 10^−01^	4.97 × 10^−01^	7.06 × 10^−02^	7.55 × 10^−02^	1.93 × 10^−01^	2.99 × 10^−02^	4.31 × 10^−01^	1.13 × 10^01^
$f_{4}$	Avg	1.39 × 10^−78^	4.02 × 10^−59^	1.11 × 10^−120^	7.05 × 10^−132^	2.15 × 10^−142^	1.35 × 10^−14^	6.28 × 10^00^	4.35 × 10^01^	2.45 × 10^02^
	Std	7.00 × 10^−79^	2.62 × 10^−59^	1.07 × 10^−120^	9.97 × 10^−132^	3.04 × 10^−142^	3.69 × 10^−16^	3.79 × 10^00^	4.73 × 10^00^	1.21 × 10^02^
$f_{5}$	Avg	2.10 × 10^−39^	8.31 × 10^−26^	9.67 × 10^−34^	1.06 × 10^−38^	2.20 × 10^−51^	1.13 × 10^−14^	1.98 × 10^−01^	6.46 × 10^00^	9.92 × 10^00^
	Std	1.18 × 10^−39^	6.06 × 10^−26^	1.36 × 10^−33^	1.41 × 10^−38^	3.11 × 10^−51^	5.10 × 10^−16^	3.79 × 10^−02^	6.16 × 10^−01^	1.68 × 10^00^
$f_{6}$	Avg	4.51 × 10^01^	4.62 × 10^01^	4.71 × 10^01^	4.44 × 10^01^	4.17 × 10^01^	4.89 × 10^01^	2.81 × 10^02^	5.23 × 10^04^	7.38 × 10^03^
	Std	8.40 × 10^−01^	1.77 × 10^−02^	6.93 × 10^−01^	5.34 × 10^−01^	1.19 × 10^00^	4.18 × 10^−02^	6.17 × 10^01^	1.85 × 10^03^	7.35 × 10^03^
$f_{7}$	Avg	5.46 × 10^−23^	9.34 × 10^−18^	2.51 × 10^−44^	7.58 × 10^−53^	3.99 × 10^−50^	1.44 × 10^−14^	3.42 × 10^01^	8.20 × 10^04^	3.97 × 10^04^
	Std	5.32 × 10^−23^	1.21 × 10^−17^	3.29 × 10^−53^	1.01 × 10^−52^	5.64 × 10^−50^	1.62 × 10^−16^	4.71 × 10^00^	7.45 × 10^03^	6.11 × 10^03^
$f_{8}$	Avg	1.78 × 10^−76^	2.70 × 10^−56^	5.70 × 10^−77^	1.38 × 10^−137^	6.39 × 10^−138^	1.18 × 10^−14^	3.90 × 10^−01^	8.48 × 10^00^	4.91 × 10^00^
	Std	2.08 × 10^−76^	3.11 × 10^−56^	4.41 × 10^−77^	9.54 × 10^−138^	8.80 × 10^−138^	6.86 × 10^−16^	1.10 × 10^−01^	2.19 × 10^−01^	1.69 × 10^00^
$f_{9}$	Avg	−1.07 × 10^04^	−1.20 × 10^04^	−8.98 × 10^03^	−1.02 × 10^04^	−1.33 × 10^04^	−6.68 × 10^03^	−4.30 × 10^03^	2.19 × 10^03^	5.02 × 10^03^
	Std	1.45 × 10^03^	7.64 × 10^02^	6.41 × 10^02^	5.08 × 10^02^	1.29 × 10^03^	3.47 × 10^02^	1.19 × 10^04^	6.73 × 10^03^	1.32 × 10^04^
$f_{10}$	Avg	0.00 × 10^00^	0.00 × 10^00^	0.00 × 10^00^	0.00 × 10^00^	0.00 × 10^00^	0.00 × 10^00^	8.08 × 10^01^	4.55 × 10^02^	8.16 × 10^01^
	Std	0.00 × 10^00^	0.00 × 10^00^	0.00 × 10^00^	0.00 × 10^00^	0.00 × 10^00^	0.00 × 10^00^	9.20 × 10^00^	1.02 × 10^01^	4.82 × 10^00^
$f_{11}$	Avg	1.48 × 10^−14^	2.27 × 10^−14^	7.66 × 10^−15^	1.76 × 10^−16^	8.06 × 10^−15^	6.55 × 10^−12^	2.79 × 10^00^	5.89 × 10^00^	5.14 × 10^00^
	Std	3.96 × 10^−16^	6.60 × 10^−16^	4.71 × 10^−16^	3.51 × 10^−15^	7.12 × 10^−16^	4.63 × 10^−12^	7.86 × 10^−01^	2.81 × 10^−01^	8.84 × 10^−01^
$f_{12}$	Avg	0.00 × 10^00^	0.00 × 10^00^	0.00 × 10^00^	0.00 × 10^00^	0.00 × 10^00^	7.75 × 10^−01^	6.86 × 10^−01^	9.24 × 10^00^	3.13 × 10^01^
	Std	0.00 × 10^00^	0.00 × 10^00^	0.00 × 10^00^	0.00 × 10^00^	0.00 × 10^00^	3.14 × 10^−02^	3.50 × 10^−02^	6.48 × 10^−01^	1.90 × 10^01^
$f_{13}$	Avg	1.92 × 10^−01^	8.52 × 10^−08^	9.99 × 10^−02^	1.13 × 10^−02^	3.90 × 10^−08^	3.00 × 10^−01^	9.56 × 10^−01^	5.24 × 10^00^	1.42 × 10^01^
	Std	1.08 × 10^−02^	3.68 × 10^−08^	0.00 × 10^00^	7.36 × 10^−03^	4.98 × 10^−08^	3.78 × 10^−04^	4.17 × 10^−02^	2.32 × 10^−01^	3.56 × 10^00^
$f_{14}$	Avg	0.00 × 10^00^	0.00 × 10^00^	0.00 × 10^00^	0.00 × 10^00^	0.00 × 10^00^	1.37 × 10^00^	5.47 × 10^−02^	3.05 × 10^00^	3.72 × 10^00^
	Std	0.00 × 10^00^	0.00 × 10^00^	0.00 × 10^00^	0.00 × 10^00^	0.00 × 10^00^	1.94 × 10^00^	6.06 × 10^−03^	1.56 × 10^−01^	1.14 × 10^00^
$f_{15}$	Avg	1.92 × 10^−01^	8.52 × 10^−08^	9.99 × 10^−02^	1.13 × 10^−02^	3.90 × 10^−08^	3.00 × 10^−01^	9.56 × 10^−01^	5.24 × 10^00^	1.42 × 10^01^
	Std	1.08 × 10^−02^	3.68 × 10^−08^	0.00 × 10^00^	7.36 × 10^−03^	4.98 × 10^−08^	3.78 × 10^−04^	4.17 × 10^−02^	2.32 × 10^−01^	3.56 × 10^00^
$f_{16}$	Avg	6.16 × 10^−38^	2.98 × 10^−07^	5.17 × 10^−40^	4.76 × 10^−80^	8.64 × 10^−66^	1.72 × 10^−15^	3.89 × 10^00^	3.91 × 10^01^	1.20 × 10^00^
	Std	8.63 × 10^−38^	4.21 × 10^−07^	4.38 × 10^−80^	6.73 × 10^−40^	1.22 × 10^−65^	1.31 × 10^−16^	2.43 × 10^−01^	2.02 × 10^−01^	5.86 × 10^−01^
$f_{17}$	Avg	−2.06 × 10^00^	−2.06 × 10^00^	−2.06 × 10^00^	−2.06 × 10^00^	−2.06 × 10^00^	−2.06 × 10^00^	−2.06 × 10^00^	−2.06 × 10^00^	−2.06 × 10^00^
	Std	0.00 × 10^00^	0.00 × 10^00^	0.00 × 10^00^	0.00 × 10^00^	0.00 × 10^00^	0.00 × 10^00^	0.00 × 10^00^	0.00 × 10^00^	0.00 × 10^00^
$f_{18}$	Avg	−1.00 × 10^00^	−1.00 × 10^00^	−1.00 × 10^00^	−1.00 × 10^00^	−1.00 × 10^00^	−9.96 × 10^−01^	−1.00 × 10^00^	−1.00 × 10^00^	2.91 × 10^−01^
	Std	0.00 × 10^00^	0.00 × 10^00^	0.00 × 10^00^	0.00 × 10^00^	0.00 × 10^00^	1.24 × 10^−03^	0.00 × 10^00^	0.00 × 10^00^	9.13 × 10^−01^
$f_{19}$	Avg	−9.60 × 10^02^	−9.60 × 10^02^	−9.60 × 10^02^	−9.60 × 10^02^	−9.60 × 10^02^	−9.60 × 10^02^	−9.38 × 10^02^	−9.60 × 10^02^	−9.28 × 10^02^
	Std	0.00 × 10^00^	0.00 × 10^00^	0.00 × 10^00^	0.00 × 10^00^	0.00 × 10^00^	2.74 × 10^−02^	3.07 × 10^01^	0.00 × 10^00^	2.93 × 10^01^
$f_{20}$	Avg	−1.92 × 10^01^	−1.92 × 10^01^	−1.92 × 10^01^	−1.92 × 10^01^	−1.92 × 10^01^	−1.92 × 10^01^	−1.92 × 10^01^	−1.92 × 10^01^	−1.92 × 10^01^
	Std	0.00 × 10^00^	0.00 × 10^00^	0.00 × 10^00^	0.00 × 10^00^	0.00 × 10^00^	3.65 × 10^−03^	0.00 × 10^00^	0.00 × 10^00^	0.00 × 10^00^
$f_{21}$	Avg	2.93 × 10^−01^	2.93 × 10^−01^	2.93 × 10^−01^	2.93 × 10^−01^	2.93 × 10^−01^	2.93 × 10^−01^	2.93 × 10^−01^	2.93 × 10^−01^	2.93 × 10^−01^
	Std	0.00 × 10^00^	0.00 × 10^00^	0.00 × 10^00^	0.00 × 10^00^	0.00 × 10^00^	8.16 × 10^−06^	0.00 × 10^00^	0.00 × 10^00^	2.12 × 10^−04^
$f_{22}$	Avg	1.59 × 10^−09^	6.26 × 10^−09^	2.06 × 10^−09^	1.45 × 10^−09^	3.58 × 10^−08^	1.22 × 10^−05^	1.35 × 10^−31^	1.35 × 10^−31^	7.62 × 10^−16^
	Std	2.89 × 10^−10^	3.99 × 10^−09^	7.96 × 10^−10^	1.10 × 10^−09^	4.62 × 10^−08^	6.84 × 10^−06^	0.00 × 10^00^	0.00 × 10^00^	5.11 × 10^−16^
$f_{23}$	Avg	0.00 × 10^00^	0.00 × 10^00^	0.00 × 10^00^	0.00 × 10^00^	0.00 × 10^00^	5.92 × 10^−16^	0.00 × 10^00^	0.00 × 10^00^	2.10 × 10^−03^
	Std	0.00 × 10^00^	0.00 × 10^00^	0.00 × 10^00^	0.00 × 10^00^	0.00 × 10^00^	3.77 × 10^−16^	0.00 × 10^00^	0.00 × 10^00^	2.39 × 10^−03^
$f_{24}$	Avg	−1.87 × 10^02^	−1.87 × 10^02^	−1.87 × 10^02^	−1.87 × 10^02^	−1.87 × 10^02^	−1.87 × 10^02^	−1.87 × 10^02^	−1.87 × 10^02^	−1.87 × 10^02^
	Std	0.00 × 10^00^	2.22 × 10^−03^	8.82 × 10^−03^	0.00 × 10^00^	1.76 × 10^03^	1.30 × 10^−02^	0.00 × 10^00^	0.00 × 10^00^	0.00 × 10^00^

**Table 8:** Wilcoxon rank test order.

Functions	Rank
$f_{1}$	E-GWO(1)<EWB-GWO(2)<GWO(3)<B-GWO(4)<ABC(5)<W-GWO(6)<PSO(7.5)≈GA(7.5)<BOA(9)
$f_{2}$	EWB-GWO(1)<B-GWO(2)<E-GWO(3)<W-GWO(4)<GWO(5)<BOA(6)<PSO(7)<GA(8)<ABC(9)
$f_{3}$	EWB-GWO(1)<GWO(2)<E-GWO(3)<B-GWO(4)<PSO(5)<ABC(6)<W-GWO(7)<BOA(8)<GA(9)
$f_{4}$	EWB-GWO(1)<E-GWO(2)<W-GWO(3)<GWO(4)<B-GWO(5)<BOA(6)<PSO(7)<ABC(8)<GA(9)
$f_{5}$	EWB-GWO(1)<GWO(2)<E-GWO(3)<W-GWO(4)<B-GWO(5)<BOA(6)<PSO(7)<ABC(8)<GA(9)
$f_{6}$	EWB-GWO(1)<E-GWO(2)<GWO(3)<B-GWO(4)<W-GWO(5)<BOA(6)<PSO(7)<GA(8)<ABC(9)
$f_{7}$	E-GWO(1)<EWB-GWO(2)<W-GWO(3)<GWO(4)<B-GWO(5)<BOA(6)<PSO(7)<GA(8)<ABC(9)
$f_{8}$	EWB-GWO(1)<E-GWO(2)<W-GWO(3)<GWO(4)<B-GWO(5)<BOA(6)<PSO(7)<GA(8)<ABC(9)
$f_{9}$	EWB-GWO(1)<B-GWO(2)<GWO(3)<E-GWO(4)<W-GWO(5)<BOA(6)<PSO(7)<ABC(8)<GA(9)
$f_{10}$	EWB-GWO(3.5)≈E-GWO(3.5)≈B-GWO(3.5)≈W-GWO(3.5)≈GWO(3.5)≈BOA(3.5)<PSO(7)<GA(8)<ABC(9)
$f_{11}$	E-GWO(1)<W-GWO(2)<EWB-GWO(3)<GWO(4)<B-GWO(5)<BOA(6)<PSO(7)<GA(8)<ABC(9)
$f_{12}$	EWB-GWO(3)≈B-GWO(3)≈E-GWO(3)≈W-GWO(3)≈GWO(3)<PSO(6)<BOA(7)<ABC(8)<GA(9)
$f_{13}$	EWB-GWO(1)<B-GWO(2)<E-GWO(3)<W-GWO(4)<GWO(5)<BOA(6)<PSO(7)<ABC(8)<GA(9)
$f_{14}$	EWB-GWO(3)≈EWB-GWO(3)≈B-GWO(3)≈E-GWO(3)≈W-GWO(3)≈GWO(3)<PSO(6)<BOA(7)<ABC(8)<GA(9)
$f_{15}$	EWB-GWO(1)<B-GWO(2)<E-GWO(3)<W-GWO(4)<GWO(5)<BOA(6)<PSO(7)<ABC(8)<GA(9)
$f_{16}$	E-GWO(1)<EWB-GWO(2)<W-GWO(3)<GWO(4)<BOA(5)<B-GWO(6)<GA(7)<PSO(8)<ABC(9)
$f_{17}$	EWB-GWO(5)≈B-GWO(5)≈E-GWO(5)≈W-GWO(5)≈GWO(5)≈BOA(5)≈PSO(5)≈ABC(5)≈GA(5)
$f_{18}$	EWB-GWO(4)≈B-GWO(4)≈E-GWO(4)≈W-GWO(4)≈GWO(4)≈PSO(4)≈ABC(4)<BOA(8)<GA(9)
$f_{19}$	EWB-GWO(4)≈B-GWO(4)≈E-GWO(4)≈W-GWO(4)≈GWO(4)≈BOA(4)≈ABC(4)≈PSO(8)<GA(9)
$f_{20}$	EWB-GWO(5)≈B-GWO(5)≈E-GWO(5)≈W-GWO(5)≈GWO(5)≈BOA(5)≈PSO(5)≈ABC(5)≈GA(5)
$f_{21}$	EWB-GWO(5)≈B-GWO(5)≈E-GWO(5)≈W-GWO(5)≈GWO(5)≈BOA(5)≈PSO(5)≈ABC(5)≈GA(5)
$f_{22}$	PSO(1.5)≈ABC(1.5)<GA(3)<E-GWO(4)<GWO(5)<W-GWO(6)<B-GWO(7)<EWB-GWO(8)<BOA(9)
$f_{23}$	EWB-GWO(4)≈E-GWO(4)≈W-GWO(4)≈B-GWO(4)≈GWO(4)≈PSO(4)≈ABC(4)<BOA(8)<GA(9)
$f_{24}$	EWB-GWO(5)≈B-GWO(5)≈E-GWO(5)≈W-GWO(5)≈GWO(5)≈BOA(5)≈PSO(5)≈ABC(5)≈GA(5)

**Table 9:** Friedman test statistical results of nine algorithms.

Functions	Sum of Squares	Degree of Freedom	Mean Squares	*p*-Value
$f_{1}$	119.25	8	14.9063	0.0429
$f_{2}$	116	8	15.47	0.0407
$f_{3}$	100	8	13.33	0.1009
$f_{4}$	120	8	15	0.0424
$f_{5}$	120	8	15	0.0424
$f_{6}$	121	8	16.375	0.0404
$f_{7}$	117	8	14.625	0.0485
$f_{8}$	119	8	14.875	0.0443
$f_{9}$	88	8	11	0.1635
$f_{10}$	84	8	15.81	0.0452
$f_{11}$	102	8	13.6	0.0928
$f_{12}$	99	8	12.375	0.0447
$f_{13}$	109	8	13.625	0.0489
$f_{14}$	97	8	15.52	0.0498
$f_{15}$	109	8	14.53	0.0489
$f_{16}$	112	8	14	0.0405
$f_{17}$	0	8	0	1
$f_{18}$	64	8	8	0.0424
$f_{19}$	68.25	8	8.53125	0.0662
$f_{20}$	9	8	1.125	0.4335
$f_{21}$	16	8	2	0.4335
$f_{22}$	116	8	14.5	0.0485
$f_{23}$	64	8	8	0.0424
$f_{24}$	25	8	3.125	0.4335

**Table 10:** Map information selected for the dataset.

Map Name		Map Size (m)	Number of Obstacles	Max Length (m)
City Map	Boston	256 × 256	48,286	363.4579
	Denver	256 × 256	48,202	375.5584
	Milan	256 × 256	47,331	362.6001
	Moscow	256 × 256	48,101	362.9137
	New York	256 × 256	48,299	362.9015
	Shanghai	256 × 256	48,708	347.7005
Warcraft III	battleground	256 × 256	23,067	231.8498
	harvestmoon	256 × 256	28,648	283.9665
	duskwood	256 × 256	31,807	215.9625
	frostsabre	256 × 256	22,845	223.2813
	golemsinthemist	256 × 256	27,707	259.4772
	plainsofsnow	256 × 256	26,458	211.6589

**Table 11:** Experimental results of path planning under city maps.

Map	Algorithm Type	Path Length (m)	Total Turning Angle of the Path (°)	Calculation Time (s)	Memory Usage (kb)	Number of Nodes
Boston	A*	267.8772	990.0000	7.138933	1852.9219	19
	JPS	267.8772	135.0000	0.3088	1059.2578	19
	RRT	408.5665	3338.5940	186.8712	572.4229	94
	Theta*	256.6033	49.9276	7.3710	1899.1718	4
	BOA	297.7898	120.9959	1.1564	1564.0421	4
	EWB-GWO	250.5955	48.1721	1.8218	1561.8130	4
Denver	A*	276.1615	1170.0000	11.2521	1951.9844	219
	JPS	276.1615	315.0000	0.4641	1077.7109	33
	RRT	436.7546	3034.6210	61.4231	533.5322	100
	Theta*	258.3104	52.7972	11.5335	1102.2732	6
	BOA	270.1912	270.1927	2.5235	1585.8192	4
	EWB-GWO	258.8289	62.9828	2.7950	1582.1092	4
Milan	A*	270.8356	1620.0000	13.6309	2035.2344	229
	JPS	270.8356	495.0000	0.7813	1132.5547	29
	RRT	407.7956	3108.8480	142.7865	558.5557	93
	Theta*	258.1860	38.9810	13.9132	1190.1270	6
	BOA	262.6911	44.6869	2.1344	1578.7026	4
	EWB-GWO	255.9445	35.6227	1.9219	1574.6176	3
Moscow	A*	300.2447	1530.0000	13.0021	1710.5781	229
	JPS	298.2447	405.0000	0.6125	1089.5781	39
	RRT	405.3163	3039.8970	132.5529	555.3838	95
	Theta*	289.0161	83.1341	13.2845	1785.5801	6
	BOA	290.7984	46.5618	5.8887	1537.7820	3
	EWB-GWO	289.8957	46.3224	5.0168	1568.8093	3
New York	A*	310.4335	3960.0000	18.6781	2478.4688	257
	JPS	310.4335	675.0000	0.5872	1103.343	30
	RRT	368.6966	3173.0940	110.6314	548.5400	87
	Theta*	285.4566	44.7358	19.8253	2501.4208	10
	BOA	283.3957	39.4442	1.4239	1540.5	4
	EWB-GWO	283.0304	34.7131	1.5010	1502.2	3
Shanghai	A*	300.6173	2160.0000	19.6747	2105.7344	236
	JPS	300.6173	495.0000	0.5586	1061.5781	26
	RRT	366.1955	2851.8291	48.8274	529.2666	86
	Theta*	282.7505	54.8031	19.9811	2180.3429	5
	BOA	302.7648	102.0262	3.1015	1554.8193	4
	EWB-GWO	282.6029	24.0939	2.8359	1555.7849	3

**Table 12:** Experimental results of path planning under game maps.

Map	Algorithm Type	Path Length (m)	Total Turning Angle of the Path (°)	Calculation Time (s)	Memory Usage (kb)	Number of Nodes
battleground	A*	176.7817	630.0000	5.2977	2221.2031	154
	JPS	176.7817	171.6604	1.0484	1870.9609	27
	RRT	222.3185	1683.270	80.1531	522.3135	52
	Theta*	171.6604	89.7732	5.6248	2301.4532	6
	BOA	174.2078	93.3885	2.6984	1579.2133	4
	EWB-GWO	170.6907	90.1226	2.6910	15,787.7810	4
harvestmoon	A*	191.0955	1080.0000	4.6075	2089.2813	165
	JPS	191.0955	1035.0000	0.7749	1705.7109	43
	RRT	263.8107	2049.297	98.1736	521.9932	61
	Theta*	186.7259	656.6668	4.8306	2020.3452	114
	BOA	182.8787	66.8938	2.9988	1556.7690	4
	EWB-GWO	183.7229	63.8977	3.0292	1554.1269	5
duskwood	A*	216.7766	1170.0000	0.5311	1812.1250	165
	JPS	216.7766	585.0000	8.3422	1579.6875	39
	RRT	338.8771	2269.2270	45.0792	673.2666	78
	Theta*	216.7398	102.0471	8.5788	1880.4324	11
	BOA	217.2969	104.7780	5.4616	1557.4368	4
	EWB-GWO	215.2443	95.2199	4.9328	1555.6790	4
frostsabre	A*	234.5046	2160.0000	7.1108	2303.5469	179
	JPS	232.5046	1125.0000	1.1715	1891.9922	63
	RRT	285.8457	2162.9054	76.6554	522.1338	67
	Theta*	211.1609	74.6927	7.3739	1384.3443	12
	BOA	230.4921	46.5942	5.0096	1576.9384	4
	EWB-GWO	228.7364	46.4878	4.0595	1574.6754	4
golemsinthemist	A*	199.4508	1170.0000	11.2086	2184.5938	163
	JPS	199.4508	225.0000	1.1425	1718.7734	27
	RRT	251.4762	2157.0730	95.4560	545.0088	59
	Theta*	186.2028	110.7750	11.7007	2231.4853	10
	BOA	196.0223	89.0916	5.6584	1558.1250	4
	EWB-GWO	196.7637	81.6472	3.7590	1556.6089	5
plainsofsnow	A*	209.5391	3330.0000	10.7168	2483.0938	188
	JPS	209.5391	585.0000	0.8499	1822.6953	24
	RRT	314.9667	2852.405	99.2433	587.4385	72
	Theta*	192.8992	82.9728	11.0657	2520.2432	7
	BOA	194.1490	60.2447	5.7059	1561.8362	3
	EWB-GWO	192.8901	60.1029	5.2394	1560.0021	3

## Data Availability

Publicly available datasets were analyzed in this study. This data can be found here: [https://movingai.com/, accessed on 10 January 2023].
